# Light-activated photodeformable supramolecular dissipative self-assemblies

**DOI:** 10.1038/s41467-022-30969-2

**Published:** 2022-06-09

**Authors:** Xu-Man Chen, Wei-Jie Feng, Hari Krishna Bisoyi, Shu Zhang, Xiao Chen, Hong Yang, Quan Li

**Affiliations:** 1 Institute of Advanced Materials, School of Chemistry and Chemical Engineering, and Jiangsu Province Hi-Tech Key Laboratory for Biomedical Research, Southeast University, Nanjing, 211189 China; 2 Advanced Materials and Liquid Crystal Institute and Chemical Physics Interdisciplinary Program, Kent State University, Kent, OH 44242 USA

**Keywords:** Molecular self-assembly, Organic molecules in materials science, Self-assembly

## Abstract

Dissipative self-assembly, one of fundamentally important out-of-equilibrium self-assembly systems, can serve as a controllable platform to exhibit temporal processes for various non-stimulus responsive properties. However, construction of light-fueled dissipative self-assembly structures with transformable morphology to modulate non-photoresponsive properties remains a great challenge. Here, we report a light-activated photodeformable dissipative self-assembly system in aqueous solution as metastable fluorescent palette. Zwitterionic sulfonato-merocyanine is employed as a light-induced amphiphile to co-assemble with polyethyleneimine after light irradiation. The formed spherical nanoparticles spontaneously transform into cuboid ones in the dark with simultaneous variation of the particle sizes. Then the two kinds of nanoparticles can reversibly interconvert to each other by periodical light irradiation and thermal relaxation. Furthermore, after loading different fluorophores exhibiting red, green, blue emissions and their mixtures, all these fluorescent dissipative deformable nanoparticles display time-dependent fluorescence variation with wide range of colors. Owing to the excellent performance of photodeformable dissipative assembly platform, the light-controlled fluorescence has achieved a 358-fold enhancement. Therefore, exposing the nanoparticles loaded with fluorophores to light in a spatially controlled manner allows us to draw multicolored fluorescent images that spontaneously disappeared after a specific period of time.

## Abstract (web summary)

Dissipative self-assembly can serve as a controllable platform to exhibit temporal processes for various non-stimulus responsive properties but construction of light-fueled dissipative self-assembly structures with transformable morphology to modulate non-photoresponsive properties remains a challenge. Here, the authors report a light-activated photodeformable dissipative self-assembly system in aqueous solution as metastable fluorescent platform.

## Introduction

Living organisms are natural dissipative self-assembly systems (DSAs) with complex assembly structures^1,2^. Some of them have the ability to adapt their morphologies to achieve advanced functions such as the deformation movement of macrophages for phagocytosis^3,4^. In order to realize these functions, they usually consume energy to deform into their functional assembled states. Such phenomena have raised much attention toward the development of artificial deformable supramolecular DSAs^5–11^. Various energies have been employed for deformation of self-assemblies including thermal energy, sound energy and chemical energy^12–16^, while light energy has unique advantages due to its clean, noninvasive, remote controllable properties with precise location and on-demand tunability^17,18^. Although some developed light-controlled supramolecular self-assemblies bear different light-responsive moieties including azobenzenes^19–21^, spiropyrans^22,23^, and diarylethenes^24,25^, photodeformable supramolecular DSA is particularly important yet rarely reported before.

Construction of light-controlled platforms to modulate non-photoresponsive properties is one of great issues for light-responsive systems and has attracted great interests recently^26–28^. In these systems, photoresponsive platforms and their loading functional moieties perform their duties respectively, and the properties of functional moieties can be tuned by the light-induced variation of photoresponsive platforms^29^. In comparison to functional self-assemblies that are inherently photoresponsive, using photoresponsive assembly platforms to control non-photoresponsive functions have several advantages: photoresponsive platforms and other functional systems can be obtained separately, which prevents the cumbersome synthesis to bind them; photoresponsive and other functional moieties would not restrict each other on their performance; photoresponsive platforms can widely adapt to various kinds of functional systems, even hybrid functional systems, for endowing them light-controllable property.

Fluorescence, one of typical luminescence, is extensively used on imaging^30,31^, diagnosis^32,33^, display^34,35^, encryption^36,37^ and anti-counterfeiting^38,39^. Diverse fluorescent dyes with stimuli-responsive properties have been studied^40,41^, while light-controlled fluorescent dyes are more superior for smart luminescent materials^42^. However, the development of light-controlled fluorescent system still depends on covalently modifying photoresponsive moieties with fluorophores. Nevertheless, such strategies not only require cumbersome synthesis, but also limit the performance of light-controlled fluorescence. Although structural optimization^43^ and application of host-guest complexation^44^ are two strategies for inherent photoresponsive fluorescent dyes, photoresponsive assembly platforms is potential to be a more effective way to improve light-controlled fluorescence systems.

**Fig. 1:**
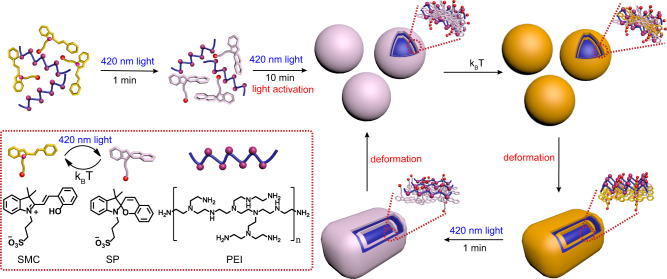
Schematic illustration of the light-activated supramolecular DSAs. Light-activation process for dissipative self-assembly (initial two steps), and reversibly photodeformable dissipative self-assembly under light irradiation/thermal relaxation cycles (following steps in the loop). The schematic and chemical structures of SMC, SP, and PEI are shown in the dashed box.

Previously, light-controlled metastable inks have been developed based on the color variation by controlling the aggregation and dissociation of gold nanoparticles, which can be used for writing self-erasing images by light^26^. This work has given us inspiration to develop light-controlled metastable fluorescent inks. Herein, we report light-activated photodeformable supramolecular DSAs as controllable platforms for diverse fluorescent dyes. Zwitterionic sulfonato-merocyanine (SMC)^45^ and polyethyleneimine (PEI)^46^ were introduced as disassembly state in aqueous solution initially, while 420 nm irradiation activated the zwitterionic SMC isomerizing into the form of spiropyran sulfonic acid for following ionization into negatively charged sulfonato-spiropyran (SP) and H^+^ (Fig. 1). After that, the amino groups from PEI received the generated H^+^ to form positively charged PEI. Owing to the strong electrostatic interaction and hydrophobic effect between negatively charged SP and positively charged PEI, they became building blocks to form transient spherical supramolecular nanoparticles. Upon removing the 420 nm irradiation, SP spontaneously started to reform SMC. However, because PEI possessed high density of positive charge, the electrostatic interaction between SP and PEI were so strong that part of SP maintained their SP form in the dark. Thus, it finally became SMC-SP-PEI ternary supramolecular co-assembly system and cuboid nanoparticles with larger size due to weaker electrostatic interaction and more rigid groups provided by SMC. Accordingly, the deformable supramolecular DSAs could be transformed reversibly between SP-PEI spherical nanoparticles and SMC-SP-PEI cuboid ones by light irradiation and thermal relaxation. Moreover, as controllable fluorescent platforms, the deformable supramolecular DSAs were further employed to load scoparone, 5-carboxyfluorescein diacetate (CFDA), and sulforhodamine 101 (Rh101) as typical fluorescent dyes with blue, green, and red emissions, for realizing light-controlled multicolor fluorescence with hundreds of times emission variation. Finally, by exposing the nanoparticles that loaded these fluorophores to light in a spatially controlled manner, we could draw metastable multicolor fluorescent images, which gradually disappeared in several minutes.

## Results

### Construction of light-activated photodeformable supramolecular DSA system

**Fig. 2:**
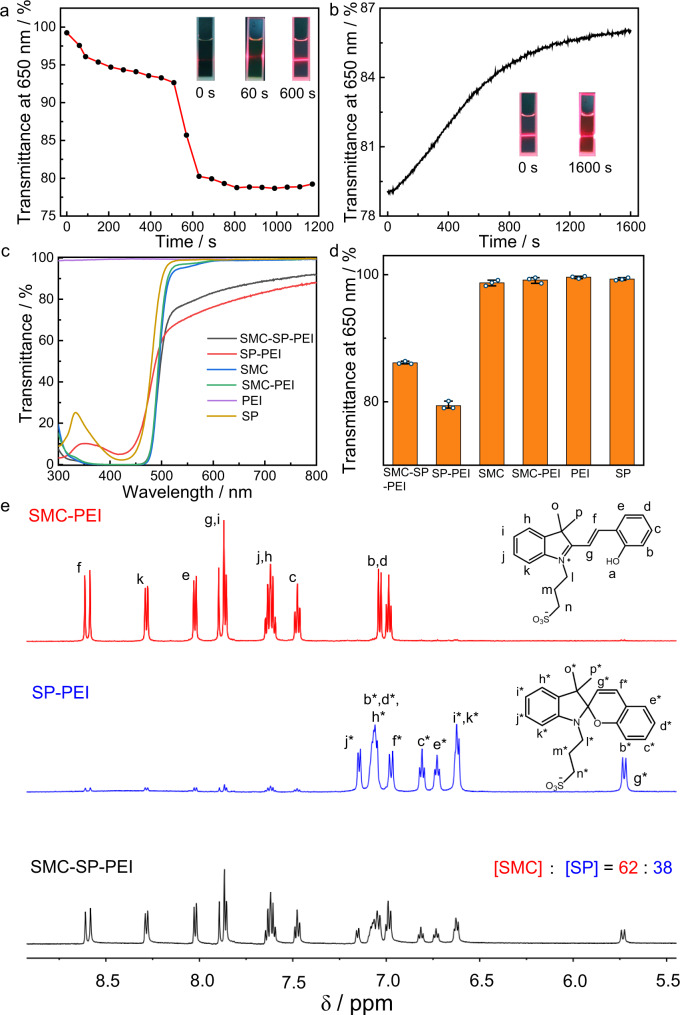
Construction of the light-activated supramolecular DSAs. **a** Decrease process of transmittance at 650 nm of SMC-PEI under 420 nm irradiation from 0 s to 1170 s. Inset: Tyndall effect photos at different irradiation time. **b** Optical transmittance at 650 nm during thermal deformation process of SP-PEI over time. Inset: Tyndall effect photos after different time in the dark. **c** Transmittance spectra of SMC-SP-PEI, SP-PEI, SMC, SMC-PEI, PEI, SP solutions. **d** Transmittance at 650 nm of **c** and the corresponding cubic error bars. *n* = 3 independent experiments, with the bar data indicating mean ± SD. ([SMC]_initial_ = 0.225 mM, [PEI] = 5 μg mL^−1^). **e** Partial ^1^H-NMR spectra (600 MHz, DMSO-d_6_) of SMC- PEI, SP-PEI, SMC-SP-PEI.

The first thing is to determine the optimal concentration of the initial SMC and PEI in aqueous solution for the supramolecular DSA. But in fact, through the optical transmittance test, we did not observe a decrease of transmittance whether we added an incremental amount of SMC to PEI (5 μg mL^–1^) or added an incremental amount of PEI to SMC (0.225 mM) without any irradiation (Supplementary Fig. 1). Then, according to the standard curves of SMC in SMC solution as well as SMC-PEI solution (Supplementary Fig. 2), the calculated isomerization efficiency of SMC in SMC-PEI solution was 92.9% under 420 nm irradiation about 30 s (Supplementary Fig. 3), which is close to the isomerization efficiency (95.5%) in only SMC solution. The pH of the SMC-PEI solution also decreased from 5.8 to 3.7 (Supplementary Fig. 4). However, the optical transmittance showed little decrease initially, then decreased obviously after continuous irradiation about 10 min, which indicated that a specific assembly process of SP and PEI happened during the 10 min under the irradiation (Fig. 2a and Supplementary Fig. 5).

Then, the critical aggregation concentration (CAC) of SP (represented by the initial concentration of SMC) in PEI was determined by investigating the optical transmittance with fixing the concentration of PEI (5 μg mL^–1^) and increasing the concentration of SP from 0.050 to 0.325 mM right after 420 nm irradiation for 15 min (Supplementary Fig. 6). The optical transmittance at 650 nm showed different linear variation with the appearance of an inflection point at 0.1521 mM, which indicated the induced CAC of SP in presence of PEI (5 μg mL^–1^) was 0.1521 mM. While ranging the concentration of PEI from 0.7 to 7.5 μg mL^–1^ in SP solution (0.225 mM), the optical transmittance at 650 nm gradually decreased before 5 μg mL^–1^ and then sharply recovered after 420 nm irradiation (Supplementary Fig. 7), which indicated the preferable mixing ratio of SP-PEI was measured as 0.225 mM SP/5 μg mL^–1^ PEI. Meanwhile, the initial 0.225 mM SP/5 μg mL^–1^ PEI showed no Tyndall effect, but an obvious Tyndall effect of SP-PEI solution appeared under 420 nm irradiation for 10 min, which indicated the formation of SP-PEI supramolecular co-assemblies (Fig. 2a).

We thought that the SP-PEI co-assemblies would disassemble back after removing 420 nm irradiation. But surprisingly, after removing the irradiation, the optical transmittance at 650 nm did not completely return to the original unassembled SMC-PEI state, but increased partially from ~79% to ~86%, which indicated that a new assembly state formed during the thermal relaxation process of SP-PEI in the dark (Fig. 2b). Meanwhile, the pH did not recover either, but only increased from 3.7 to 4.4 (Supplementary Fig. 8). Additionally, the Tyndall effect only became weaker but did not completely disappear after keeping the SP-PEI co-assemblies in the dark for 1600 s. In view of this phenomenon, we speculated that a ternary supramolecular self-assembly system emerged since part of SP isomerized back during the thermal relaxation. Thus, the absorption at 425 nm after thermal relaxation of SP in SP-PEI co-assembly was measured lower than the original absorption of SMC in SMC-PEI (Supplementary Fig. 9), which indicated that SP did not isomerize completely back to SMC during the thermal relaxation of SP-PEI co-assemblies. Accordingly, the molecular ratio of SMC to SP after thermal relaxation was calculated ~75:25, which meant that the generated self-assemblies after thermal relaxation belonged to a ternary SMC-SP-PEI supramolecular self-assembly system. The preferable concentration of SMC-SP-PEI was further determined by the same method as that of SP-PEI co-assembly. The CAC of SMC-SP (represented by the initial concentration of SMC) was 0.1581 mM, and the preferable mixing ratio of SMC-SP-PEI was 0.225 mM SMC-SP/5 μg mL^–1^ PEI, which was the same as that of SP-PEI (Supplementary Fig. 10). Therefore, the initial mixing ratio of 0.225 mM SMC /5 μg mL^–1^ PEI was preferable for all the experiments of this photoresponsive supramolecular DSA below. Dialysis experiments of SP-PEI showed little decrease of absorption <10%, which indicated that above 90% of the building blocks participated in the supramolecular DSAs (Supplementary Fig. 11). Besides, we wondered whether SMC-PEI would spontaneously assemble after enough time without any light irradiation. However, when we left 0.225 mM SMC /5 μg mL^–1^ PEI solution in the dark for as long as 10 h, the transmittance at 650 nm still kept above 99% (Supplementary Fig. 12), which indicated that there were no assemblies formed in SMC-PEI system. Overall, comparing all the optical transmittance spectra of the DSAs and any of their components, only SP-PEI and SMC-SP-PEI possessed obvious decrease at 650 nm (Fig. 2c, d). Furthermore, ^1^H-NMR spectra of SMC-PEI, SP-PEI, and SMC-SP-PEI also showed the similar light-activation and thermal relaxation process of the co-assemblies. As shown in Fig. 2e, under 420 nm irradiation for 10 min, >90% of SMCs in SMC-PEI isomerized to SPs, which agreed with the result from UV-Vis spectra. When SP-PEI was kept in the dark for 30 min, part of SPs isomerized back and the molecular ratio of SMC and SP finally reached 62: 38, which is also similar with the result from UV-Vis (Supplementary Figs. 13 and 14). Therefore, 420 nm light not only changed the assembly state between SP-PEI and SMC-SP-PEI systems, but also triggered the formation of these assemblies.

Furthermore, the factors to influence the light-activation and thermal relaxation processes were investigated. Firstly, because SMC and SP are both pH-active molecules, we wonder whether such light-activation and thermal relaxation processes are influenced in different pH conditions. To demonstrate this, the light-activation and thermal relaxation processes beginning from SMC-PEI solution in different pH conditions (pH = 1–7) were performed (Supplementary Fig. 15). When the initial pH of SMC-PEI was adjusted to 4–7, the stable interaction between SP and PEI hindered the isomerization from SP to SMC. However, when the initial pH of SMC-PEI was adjusted to 1–3, the electrostatic interaction between SP and PEI declined because of the protonation of -SO_3_^−^ group of SP to neutral -SO_3_H, so that the isomerization from SP to SMC was less hindered. Moreover, the strong interaction between SP-PEI might influence the pKa of SP. As shown in Supplementary Fig. 16, when 0.225 mM SMC (pH = 5.8) was irradiated by 420 nm light to form SP, pH decreased to 4.1, which was uncommonly >3.7 in SP-PEI solution because PEI could receive the H^+^ to reduce the pH variation during light-activation process. Therefore, we deduced that the pKa of SP increased because of the interaction between SP and PEI. Also, the formed SP-PEI co-assemblies provided confined environment to prevent the routine isomerization.

**Fig. 3:**
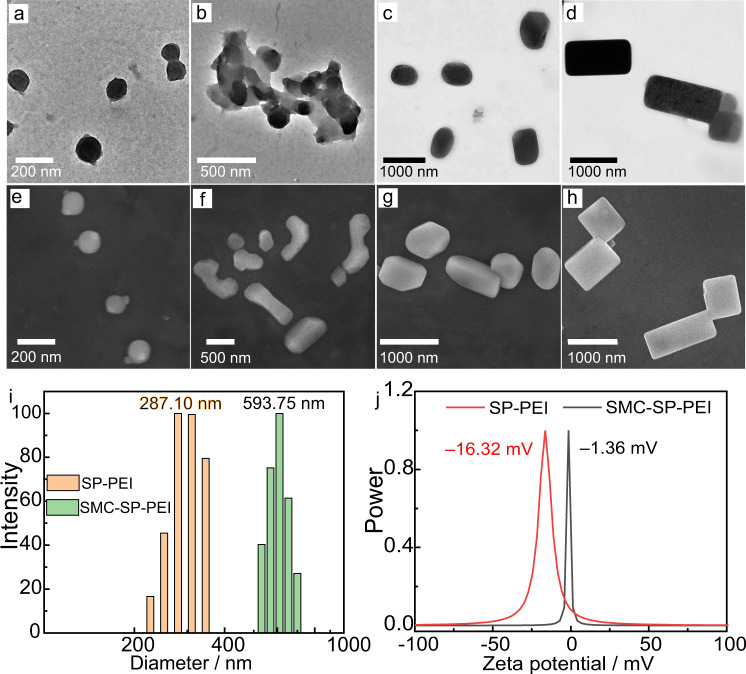
Characterization of the supramolecular DSAs. **a**–**d** TEM images of SP-PEI nanoparticles and their gradual change to SMC-SP-PEI nanoparticles. **e**–**h** SEM images of SP-PEI nanoparticles and their gradual change to SMC-SP-PEI nanoparticles. **i** DLS of SMC-SP-PEI and SP-PEI. **j** Zeta potential of SMC-SP-PEI and SP-PEI. ([SMC]_initial_ = 0.225 mM, [PEI] = 5 μg mL^−1^).

Further experiments were performed for determination of the morphological variation of the photoresponsive supramolecular DSAs. For initial SMC-PEI system without any irradiation, no obvious nanoparticles were observed in transmission electron microscopy (TEM), but after 420 nm irradiation, several spherical nanoparticles of SP-PEI assemblies were emerged with an average size of ~200 nm (Fig. 3a and Supplementary Fig. 17). After leaving the SP-PEI co-assemblies in the dark for 10 min, we observed that several spherical nanoparticles combined together (Fig. 3b), and then edges and corners appeared from the coalesced nanoparticles while keeping in the dark for another 10 min (Fig. 3c). Finally, the spherical nanoparticles spontaneously changed into cuboid ones with an enlarged average particle size of ~600 nm after keeping in the dark for 30 min (Fig. 3d), which were SMC-SP-PEI ternary co-assemblies. Meanwhile, scanning electron microscopy (SEM) images (Fig. 3e–h and Supplementary Fig. 18) was consistent with the TEM images. Dynamic light scattering (DLS) data showed that the diameter of SP-PEI was a little larger than that in SEM and TEM images (average diameter: 287.1 nm), because there was a time delay to obtain the average diameter data from DLS so that the average diameter of the very initial state of SP-PEI was hard to be investigated in time by DLS. The average diameter of SMC-SP-PEI nanoparticles was measured 593.7 nm, which was consistent with the TEM and SEM images (Fig. 3i). Because the spiropyran group of SP is a relatively flexible group, SP assemble with flexible PEI to form spherical nanoparticles. After partial SP changes into SMC during the thermal relaxation process, the electrostatic interaction decreases. Therefore, in order to ensure sufficient intermolecular forces to form SMC-SP-PEI nanoparticles, more building blocks from several spherical nanoparticles combine together, resulting larger SMC-SP-PEI nanoparticles. Additionally, because the merocyanine group of SMC possesses larger π-conjugated and rigid structure than SP for π-π stacking, the morphology of SMC-SP-PEI nanoparticles exhibit cuboid ones with edges and corners. The driving forces of photodeformable SP-PEI and SMC-SP-PEI dissipative co-assemblies are the variation of electrostatic interaction, hydrophobic interaction, and π–π stacking. Moreover, the zeta potentials of SP-PEI and SMC-SP-PEI were –16.32 and –1.36 mV, respectively, which indicated that the excess anions of SP-PEI nanoparticles were more than that of SMC-SP-PEI nanoparticles (Fig. 3j). Therefore, these results suggested the construction of light-activated photodeformable supramolecular DSAs of SP-PEI and SMC-SP-PEI.

### Dissipative properties of the photodeformable supramolecular DSAs

**Fig. 4:**
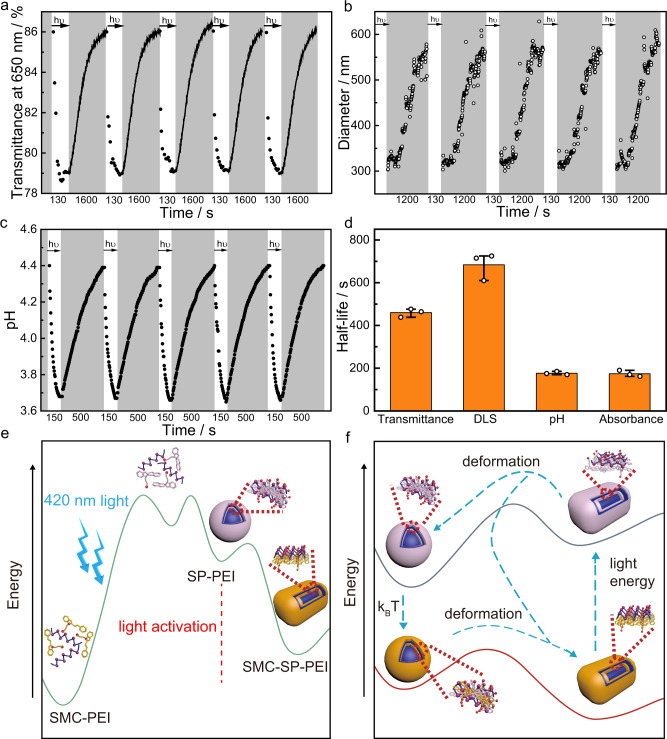
Dissipative properties of the photodeformable supramolecular DSAs. **a** Optical transmittance at 650 nm for 5 cycles between the SMC-PEI and SP-PEI dissipative process over time. The white areas represent the light-induced formation of SP-PEI nanoparticles and the gray areas represents the formation of SMC-SP-PEI nanoparticles, respectively. **b** Diameter variation during thermal deformation process from the SP-PEI to SMC-SP-PEI for 5 cycles over time measured by DLS. **c** pH variation for 5 cycles between the SMC-SP-PEI and SP-PEI dissipative process over time. The white parts represent the light-induced formation of SP-PEI and the gray parts represents the formation of SMC-SP-PEI nanoparticles, respectively. **d** Half-lives of the thermal deformation process from SP-PEI to SMC-SP-PEI exhibited by the transmittance at 650 nm, DLS, pH and absorbance at 424 nm. *n* = 3 independent experiments, with the bar data indicating mean ± SD ([SMC]_initial_ = 0.225 mM, [PEI] = 5 μg mL^−1^). **e** Schematic representation of the energy variation of the light-activation process for the photodeformable DSAs. **f** Schematic representation of the energy variation in the photodeformable dissipative process of SP-PEI and SMC-SP-PEI.

The photodeformable supramolecular DSAs between SP-PEI and SMC-SP-PEI showed good reversibility and dissipative properties after the first light activation from SMC-PEI system because of the highly stable and efficient isomerization between SMC and SP for 14 times (Supplementary Fig. 19). As shown in Fig. 4a and Supplementary Fig. 20, the optical transmittance spectra transformed between the two assembly states by 420 nm irradiation for 2 min and thermal relaxation for 30 min and repeated 10 times at 25 °C. The diameter of the nanoparticles thermally relaxed from ~300 nm to ~600 nm for ~1200 s and cycled 5 times at 25 °C, which also indicated the good reversibility of thermal relaxation from SP-PEI to SMC-SP-PEI (Fig. 4b). The pH also reversibly cycled with stable dynamics between 3.6 (SP-PEI) and 4.4 (SMC-SP-PEI) through light irradiation and thermal relaxation (Fig. 4c). It is worth noting that the thermal relaxation process from SP-PEI to SMC-SP-PEI measured by absorption and pH were significantly faster than that measured by optical transmission and diameter of nanoparticles. By absorption and pH that represented the isomerization of SPs, the half-lives of thermal relaxation were measured ~174 s and ~177 s, respectively, which were much shorter than that measured by optical transmission (~460 s) and diameter of nanoparticles (~683 s) that represented the thermal relaxation of SP-PEI nanoparticles (Fig. 4d). The difference of the half-lives demonstrated that SP firstly isomerized to SMC to form a relative higher-energy assembly state before the spontaneously deformation of the nanoparticles from spherical to cuboid ones during the thermal relaxation. Moreover, in comparison to the reversible isomerization dynamic processes between SMC and SP in SMC solution, the light-induced process of SMC-SP-PEI system was faster and the thermal relaxation of SP-PEI process was slower (Supplementary Fig. 21), which indicated that the energy of SP decreased in the photodeformable supramolecular DSAs^6,47,48^ (Fig. 4e, f). Furthermore, the kinetics of the light-induced process and the thermal relaxation process were demonstrated to be affected by optical power density and temperature, respectively (Supplementary Figs. 22 and 23). Therefore, the photodeformable supramolecular DSA system was established with a specific energy circulation.

**Fig. 5:**
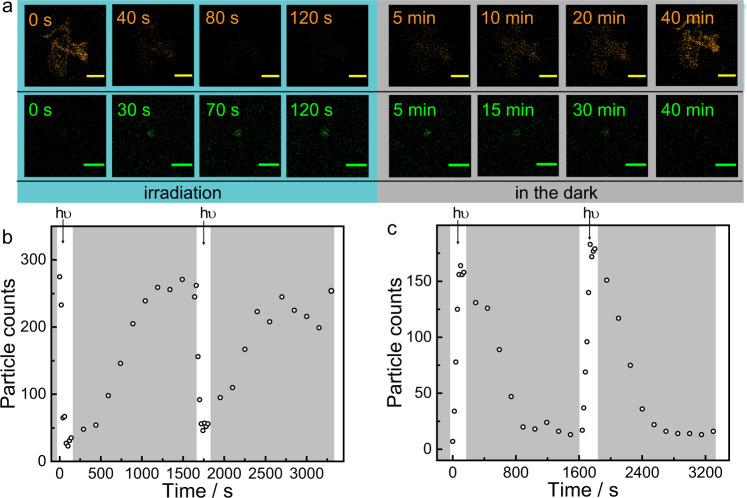
Confocal image characterization of photodeformable supramolecular DSAs. **a** Partial confocal images of the photodeformable DSA processes by loading SRB (the first row) and CFDA (the second row) (scale bar = 10 μm). **b** Total number of SRB fluorescent particle counts detected by confocal microscopy over time (measured every 20 s under 420 nm irradiaion and every 2.5 min in the dark). ([SMC]_initial_ = 0.225 mM, [PEI] = 5 μg mL^−1^, [SRB] = 0.001 mM, *λ*_ex_ = 540 nm, *λ*_em_ = 585 nm). **c** Total number of CFDA fluorescent particle counts detected by confocal microscopy over time (measured every 20 s for under 420 nm irradiaion and every 2.5 min in the dark). ([SMC]_initial_ = 0.225 mM, [PEI] = 5 μg mL^−1^, [CFDA] = 0.01 mM, *λ*_ex_ = 440 nm, *λ*_em_ = 520 nm).

Furthermore, the dissipative process of the photodeformable supramolecular DSA system was observed directly through laser scanning confocal microscopy (LSCM). Two typical fluorescent dyes, Sulforhodamine B (SRB) and carboxyfluorescein diacetate (CFDA) were loaded into the supramolecular DSA system to observe the formation of the two assembly states (Supplementary Fig. 24), respectively, because of their opposite fluorescent performance during the dissipative processes. As shown in Supplementary Fig. 25, SRB preferred fluorescing in SMC-SP-PEI nanoparticles while CFDA preferred fluorescing in SP-PEI. Then, by investigating the fluorescence after dialysis, the emission maxima of SRB (585 nm) in SMC-SP-PEI and CFDA (520 nm) in SP-PEI were calculated 98.31% and 93.46% of that before, indicating that most of these dyes were loaded in the DSAs at the given concentration (Supplementary Fig. 26). Thus, for further investigation through LSCM, SMC-SP-PEI nanoparticles could be observed by loading SRB, while fluorescent spots from LSCM images became less intense after 420 nm irradiation. By keeping in the dark for several minutes, the fluorescent spots of SMC-SP-PEI nanoparticles recovered (Fig. 5a, Supplementary Figs. 27 and 28). In contrast, CFDA-loaded SMC-SP-PEI nanoparticles showed few fluorescent spots in LSCM images, while many fluorescent spots emerged after 420 nm irradiation and further disappeared again by keeping in the dark subsequently (Fig. 5b, Supplementary Figs. 29 and 30). By counting the number of fluorescent objects, the two cyclic processes could be repeated at least twice, indicating that both SMC-SP-PEI and SP-PEI could be directly observed through LSCM during the dissipative process (Fig. 5c, and Supplementary Movies 1 and 2).

### Dynamic control of fluorophores with different colors

**Fig. 6:**
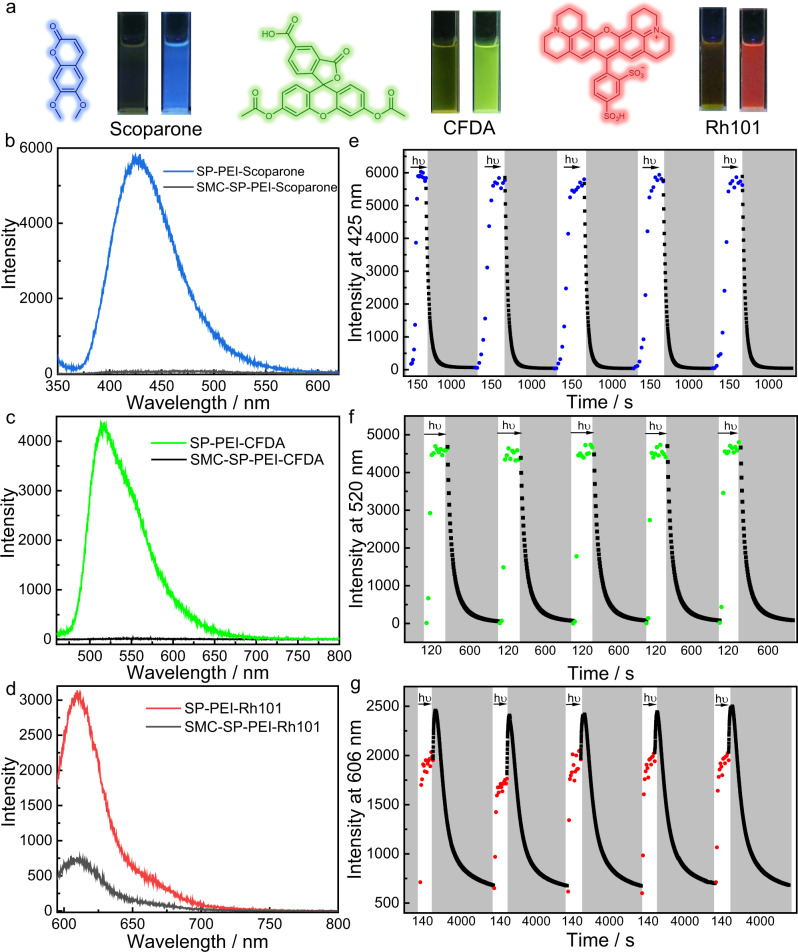
Reversible cycles and kinetics of fluorescence of scoparone, CFDA and Rh101 loaded in the supramolecular DSAs. **a** Chemical structures and their fluorescence photographs of SMC-SP-PEI (left) and SP-PEI (right) loading scoparone (blue), CFDA (green) and Rh101 (red). **b** Fluorescence emission of SMEH-SP-PEI-scoparone and SP-PEI-scoparone. **c** Fluorescence emission of SMEH-SP-PEI-CFDA and SP-PEI-CFDA. **d** Fluorescence emission of SMEH-SP-PEI-Rh101 and SP-PEI-Rh101. **e**–**g** Reversible fluorescence variation of scoparone at 425 nm, CFDA at 520 nm and Rh101 at 606 nm for 5 cycles between the SMC-SP-PEI and SP-PEI dissipative process over time. The white parts represent the light-induced formation of ASP-CS and the gray parts represents the dissociation process, respectively. ([SMC]_initial_ = 0.225 mM, [PEI] = 5 μg mL^–1^, [Scoparone] = 0.001 mM, [CFDA] = 0.01 mM, [Rh101] = 0.006 mM, *λ*_ex,Scoparone_ = 340 nm, *λ*_ex,CFDA_ = 440 nm, *λ*_ex,Rh101_ = 585 nm).

In order to achieve metastable fluorescent palette by employing the photodeformable DSAs, the capability to control the fluorescence emission of different dyes was further investigated. Thus, scoparone, sulforhodamine 101 (Rh101) as well as CFDA (Fig. 6a and Supplementary Fig. 24) were selected as representatives of blue, red, and green fluorophores to construct multicolor metastable fluorescent palette. Firstly, according to the fluorescence in SMC-SP-PEI and SP-PEI, all the three fluorophores quenched their fluorescence in SMC-SP-PEI but exhibited their strong emissions in SP-PEI upon 420 nm irradiation (Fig. 6a–d and Supplementary Movies 3–5). It is worth noting that the emission of CFDA enhanced by 358 times in SP-PEI than that in SMC-SP-PEI. To our knowledge, it is the biggest emission enhancement in light-controlled luminescent systems. In addition, the emission of scoparone and Rh101 also enhanced 114 times and 4 times in SP-PEI than that in SMC-SP-PEI, respectively. Then, by investigating the fluorescence after dialysis, the emission maxima of scoparone (425 nm) and Rh101 (606 nm) in SP-PEI were calculated to be 94.57% and 93.13% of that before, indicating that most of these dyes were loaded in the DSAs at the given concentration (Supplementary Fig. 26). Because the three fluorophores are all hydrophobic, aromatic, and electroneutral dyes, their fluorescence quenched completely through charge transfer with SMC in SMC-SP-PEI but recovered in SP-PEI due to the disappearance of charge transfer as well as better hydrophobic environment that provided by SP-PEI. In consideration that the fluorescence might be influenced by the pH variation induced by the isomerization between SMC and SP, we investigated the emission of these fluorophores in different pH. As shown in Supplementary Fig. 31, scoparone, Rh101 and SRB exhibited negligible differences of fluorescence in different pH, and the emission of CFDA quenched when the pH decreased from 7 to 3, which was opposite the performance in the photodeformable DSAs. Moreover, quantum yields of these fluorophores in SMC-SP-PEI and SP-PEI were also measured to prove the distinctions of fluorescence (Supplementary Table 1). The quantum yields of both scoparone and CFDA were measured <0.1% in SMC-SP-PEI, but in SP-PEI, their quantum yields were measured 18.59% for scoparone and 23.92% for CFDA, which indicated that the huge fluorescent variations were enabled by the DSA system. In addition, the quantum yield of Rh101 and SRB were measured 3.01% and 15.52% in SMC-SP-PEI and 14.17% and 2.75% in SP-PEI, respectively, which further demonstrated the ability of photocontrolled fluorescence of the DSA system.

Further experiments were carried out to investigate the time-dependent variation of fluorescence of the three fluorophores in the photodeformable DSAs. As shown in Supplementary Figs. 32 and 33, the emission of scoparone at 425 nm and CFDA at 520 nm in SMC-SP-PEI increased rapidly and reached stable emission in ~60 s and ~30 s upon 420 nm irradiation. After keeping in the dark, their emission gradually decreased but much more rapidly than the thermal relaxation from SP-PEI to SMC-SP-PEI. In addition, the emission of Rh101 at 606 nm also increased rapidly and reached stable emission in ~40 s upon 420 nm irradiation, but after irradiation, the emission of Rh101 continued increasing slightly in the dark first and then decreased much more slowly than the thermal relaxation from SP-PEI to SMC-SP-PEI (Supplementary Fig. 34). This phenomenon demonstrated that there are two factors that influence the dynamic control of the fluorescence in the photodeformable DSAs, one is aggregation-caused quenching and the other is hydrophobic environment induced emission enhancement. The size of SP-PEI was much smaller than SMC-SP-PEI, so that Rh101 would aggregate more tightly in SP-PEI and quenched its fluorescence. However, because SP possessed fewer electric charges than SMC, SP-PEI provided more hydrophobic environment than SMC-SP-PEI, which had more advantage for fluorescence of hydrophobic fluorophores. Because the large steric hindrance induced by the hydro-quinolizine weakened the effect of aggregation-caused quenching, Rh101 overall exhibited emission enhancement as the result of the enhanced hydrophobic environment. On the other hand, although SRB and Rh101 possessed similar molecular structure, the diethylamino groups of SRB provided smaller steric hindrance to hinder the effect of aggregation-caused quenching, so that the effect of aggregation-caused quenching became the main factor for the decrease of the emission of SRB upon 420 nm irradiation (Supplementary Fig. 35). Additionally, scoparone and CFDA are typical hydrophobic fluorophores without any charges, so that hydrophobic environment induced emission enhancement contributed more to their fluorescence enhancement upon 420 nm irradiation. Furthermore, all the fluorophores exhibited good reversibility of time-dependent fluorescent variation for at least five cycles due to the excellent reversibility between SMC-SP-PEI and SP-PEI nanoparticles as well as the photo-stability of the fluorophores (Fig. 6e–g and Supplementary Fig. 36). The half-lives of fluorescence quenching of scoparone (~44 s) and CFDA (~37 s) were much shorter than that of thermal relaxation from SP-PEI to SMC-SP-PEI, indicating that the charge transfer from SMC was highly sensitive to quench their fluorescence. As a result of the conflict between hydrophobic effect induced emission enhancement and aggregation-induced quenching, the half-life of fluorescence quenching of Rh101 (~720 s) was longer than thermal relaxation from SP-PEI to SMC-SP-PEI. Remarkably, the time-dependent fluorescence of SRB and Rh101 in the DSAs during thermal relaxation process from SP-PEI to SMC-SP-PEI both showed difference variation trends between the first 300 s and after 300 s, which was the evidence for two step (isomerization from SP to SMC then deformation) process during thermal relaxation.

**Fig. 7:**
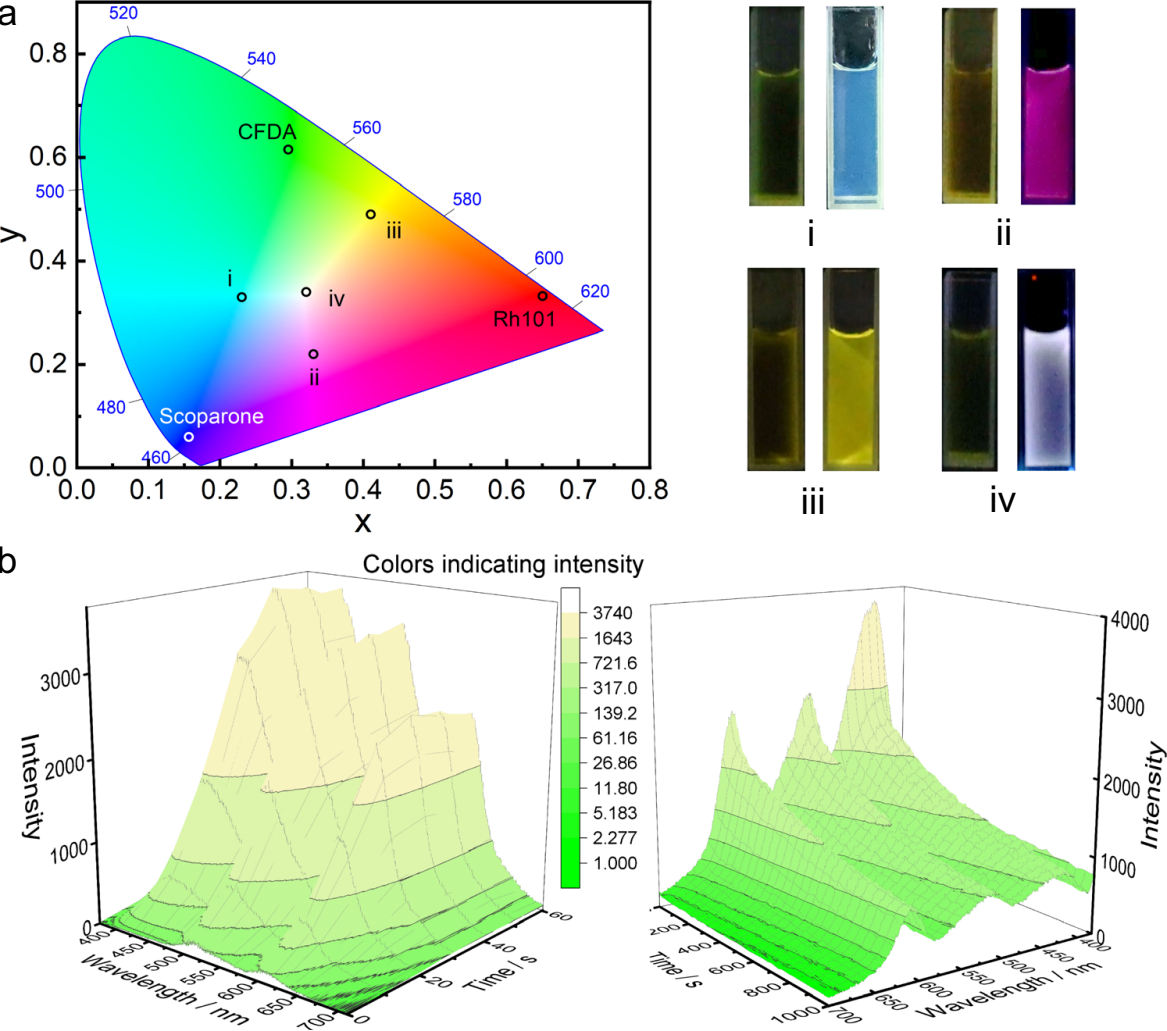
Orthogonal combination of the fluorophores loaded in the photodeformable supramolecular DSAs for more metastable fluorescent colors. **a** CIE 1931 images of light-induced fluorescent color sites in different fluorescent combinations and the corresponding fluorescence picture ((i) Scoparone + CFDA, (ii) Scoparone + Rh101, (iii) CFDA + Rh101, (iv) Scoparone + CFDA + Rh101). **b** 3D fluorescence spectra of light-induced process and thermal deformation process loading scoparone, CFDA and Rh101 for controllable of pure white emission. ([Scoparone] = 0.0002 mM, [CFDA] = 0.025 mM, [Rh101] = 0.004 mM) ([SMC]_initial_ = 0.225 mM, [PEI] = 5 μg mL^−1^).

We also wondered whether loading the mixture of these fluorophores in the photodeformable supramolecular DSAs could create more fluorescent colors to enrich the metastable fluorescent palette. To demonstrate this, we firstly determined the colors of these fluorophores by calculating their emission spectra in CIE 1931 image. (Fig. 7a). After loading the binary mixture of fluorophores, cyan (mixture of scoparone and CFDA), fuchsia (mixture of scoparone and Rh101) and yellow (mixture of CFDA and Rh101) were realized with metastable fluorescence induced by 420 nm irradiation and then spontaneously quenched in several minutes (Fig. 7a and Supplementary Figs. 37–39). Furthermore, when loading the ternary mixture of these fluorophores with probable concentrations (Fig. 7b), the photodeformable supramolecular DSAs achieved a pure white light emission that could also be controlled by 420 nm irradiation and thermal relaxation through the same method. Therefore, the metastable fluorescent palette system with abundant colors was constructed by employing three colors of fluorophores loaded into the photodeformable DSAs.

### Creating self-erasing multicolor fluorescent images

**Fig. 8:**
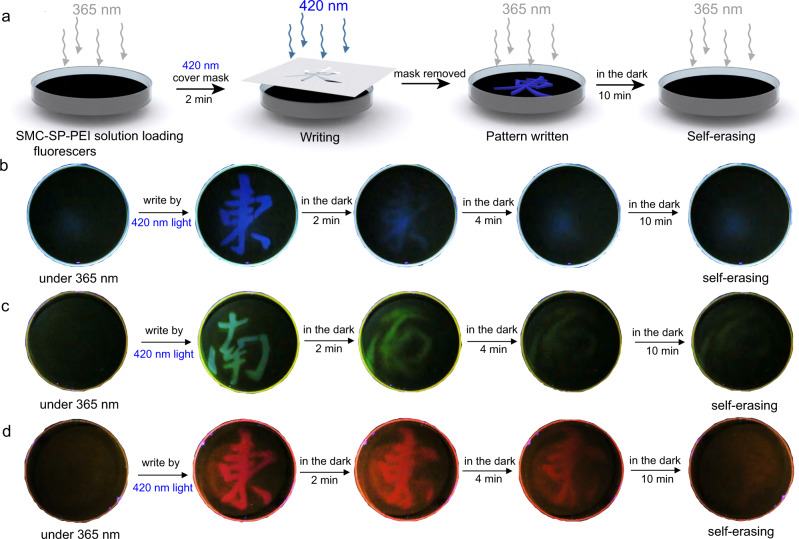
Metastable fluorescent palette for exhibiting self-erasing fluorescent patterns in petri dish. **a** Schematic representation of the 420 nm light writing and 365 nm emitting the patterns in the petri dish. **b** Photo-writing pattern and self-erasing process of the scoparone-loaded DSAs. **c** Photo-writing pattern and self-erasing process of the CFDA-loaded DSAs. **d** Photo-writing pattern and self-erasing process of the Rh101-loaded DSAs. ([SMC]_initial_ = 0.225 mM, [PEI] = 5 μg mL^−1^, [Scoparone] = 0.001 mM, [CFDA] = 0.01 mM, [Rh101] = 0.006 mM).

**Fig. 9:**
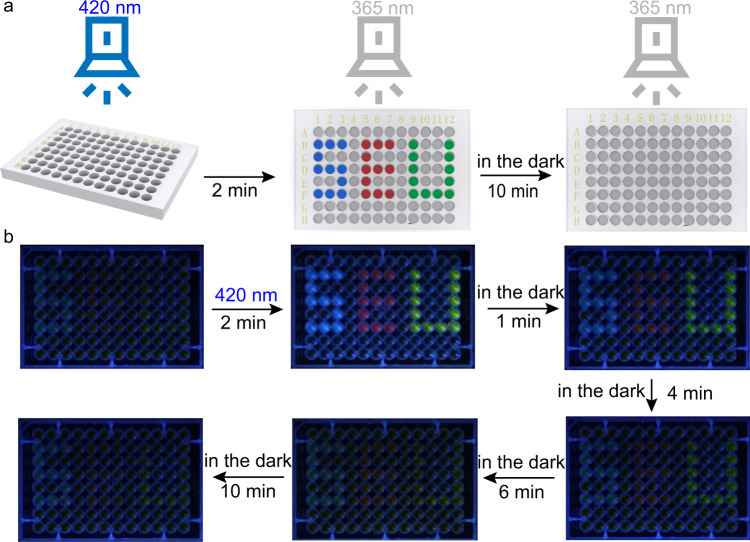
Dynamic fluorescent exhibition in 96-well plates. **a** Schematic representation of the 420 nm light writing and 365 nm emitting the patterns in the 96-well plates. **b** The gradual fluorescence change with time in the dark after 420 nm irradiation. ([SMC]_initial_ = 0.225 mM, [PEI] = 5 μg mL^−1^).

Finally, taking advantage of the fluorescence variations of the blue, green, and red fluorophores in the DSAs, we hypothesized that by irradiating a thin layer of DSA solution with fluorophores locally (e.g., through a mask), we could create fluorescent patterns that would disappear spontaneously after removing the irradiation. To demonstrate this, we filled SMC-SP-PEI solution with scoparone in a petri dish (diameter: 10 cm, thickness: 1 cm) and then irradiated with 420 nm light through a mask with Chinese character “Dong” that featured the badge of “Southeast University” (Fig. 8a, b). Blue fluorescence of character “Dong” emerged in the exposed regions, which indicated the emission enhancement by the deformation from SMC-SP-PEI to SP-PEI nanoparticles. The fluorescent pattern spontaneously faded out in several minutes in the dark, which is similar to the performance of the corresponding fluorescent dynamics. In Supplementary Fig. 40, different masks could be applied to write and erase a series of metastable fluorescent patterns (including many Chinese characters and the emblem of Southeast University, etc.) with no deterioration for at least 10 times, indicating the stability of the metastable fluorescent DSA system. Meanwhile, when loading CFDA and Rh101, the same operations could still work for the writing and self-erasing process of green and red metastable fluorescent patterns (Fig. 8c, d). In addition, when we drew the three characters “SEU” with the DSAs that loading the three fluorophores in 96-well plates, respectively, the three fluorescent characters with different colors simultaneously appeared by 420 nm irradiation and gradually disappeared in the dark (Fig. 9). All above experiments demonstrated the promising application of reversible, time-dependent, and multicolor information storage.

## Discussion

We have successfully developed a general method to construct the photodeformable supramolecular dissipative self-assembly. Taking advantage of the photo-isomerization and thermal relaxation between SMC and SP with the variation of charges and amphiphilicity, polycationic PEI assemble with them to form SMC-SP-PEI cuboid and SP-PEI spherical nanoparticles, which can transform to each other upon light irradiation and thermal relaxation with good reversibility. We envision that the strategy to induce dissipative self-assemblies of cationic polymers could be extended to other compositions, such as anionic and electroneutral polymers. Furthermore, the variation of charge and amphiphilicity for dissipative self-assembly would show similar behavior, but photoresponsive media need to possess different kinds of charges and amphiphilic groups. Small molecules are also applicable to this strategy if such photoresponsive groups are polymerized or modified on macromolecules to form macromolecular media. Therefore, a wide range of molecules can be easily induced for such dissipative self-assembly to realize photocontrollable properties in different occasions through this strategy. Moreover, the dynamics of the dissipative process can also be easily controlled when decorating SMC with various electron-withdrawing and -donating substituents. For example, modifying -NO_2_ or -COOMe groups at the para position with respect to the hydroxyphenyl would significantly prolong the dynamics of thermal relaxation process^45^. Furthermore, the sensing wavelength of the dissipative supramolecular self-assemblies can also be extended; in fact, several SMC derivatives that can be isomerized upon different wavelengths of light have been synthesized recently^49,50^.

The photodeformable supramolecular dissipative self-assembly provide suitable nanoplatforms to load fluorophores and realize multicolor time-dependent fluorescence with different dynamics by the dissipative processes. Wide range of fluorescent colors can be controlled for metastable fluorescent palette that can be used to draw colorful transient fluorescent pictures that spontaneously faded after minutes. Fluorescence and photocontrollable systems are both encryption technologies that require light sources to edit and read the information, while the spontaneous thermal relaxation induces time-dependent fluorescence that enables the transient, self-erasing fluorescent patterns to add another pathway for advanced encryption into the photocontrollable fluorescent nanosystem. Moreover, it is predictable that the dissipative nanoparticles can control other kinds of luminescence, such as phosphorescence and delayed luminescence. Beyond this, when some special catalysts (e.g., enzymes) are loaded in the photodeformable dissipative self-assemblies, we speculate that the catalytic activities of them can be modulated by light because of variation of the inner environment. In addition, more fundamentally, it seems interesting to combine the photoresponsive dissipative nanoparticles with chemical-fueled dissipative factor to construct multistage dissipative assembly systems, which can store more information or develop more advanced applications.

## Methods

### Synthesis of SMC

SMC was synthesized according to published literature^48^. 2,3,3-trimethylindolenine (1.65 g, 0.01 mmol) was firstly added into propane sultone (1.26 g, 0.01 mmol). The mixture was stirred at 90 °C for 4 h under N_2_. The purple solid was collected by filtration, washed with cold diethyl ether, and dried in vacuo to generate 2,3,3-trimethyl-1-(3-sulfonatepropyl)-3H-indolium. Then, 2,3,3-trimethyl-1-(3-sulfonatepropyl)-3H indolium (100 mg, 0.36 mmol) and 2-hydroxybenzaldehyde (48 mg, 0.39 mmol) were added into anhydrous ethanol (2 mL). The mixture was allowed to reflux overnight. The orange solid SMC was obtained by filtration. The structure was determined by ^1^H-NMR, ^13^C-NMR and HRMS (Supplementary Figs. 41, 43 and 44). ^1^H-NMR (600 MHz, DMSO-*d*_*6*_): δ 11.04 (s, 1H), 8.60 (d, *J* = 16.4 Hz, 1H), 8.29 (d, *J* = 7.3 Hz, 1H), 8.03 (d, *J* = 7.6 Hz, 1H), 7.88 (t, *J* = 11.9 Hz, 2H), 7.69–7.54 (m, 2H), 7.53–7.43 (m, 1H), 7.04 (d, *J* = 8.1 Hz, 1H), 6.99 (t, *J* = 7.5 Hz, 1H), 4.88–4.69 (m, 3H), 4.36–4.00 (m, 1H), 2.77–2.57 (m, 3H), 2.35–2.08 (m, 3H), 1.77 (s, 7H).^13^C-NMR (151 MHz, DMSO-*d*_*6*_): δ 181.16, 158.37, 148.05, 142.82, 140.28, 135.08, 129.11, 128.54, 128.52, 122.32, 120.73, 119.40, 116.00, 114.44, 110.79, 51.08, 46.84, 44.26, 25.43, 23.39. ESI-MS: m/z calculated for: C_21_H_23_NO_4_S: requires 386.14206 for [M + H]^+^, found 386.14424.

### The preparation for SP-PEI and SMC-SP-PEI photodeformable dissipative self-assemblies

SMC (0.225 mM) and PEI (5 μg mL^–1^) aqueous solution was firstly prepared at 25 °C. Then the solution was exposed under 420 nm light (15 mW cm^–2^ unless mentioned) for 10 min for light-induced assembly, SP-PEI, for further measurements. Then, SP-PEI self-assemblies was placed in the dark for ~1600 s and the self-assemblies gradually transformed to SMC-SP-PEI assemblies.

### UV-Vis spectroscopy

UV-Vis spectra and the optical transmittance were recorded in a quartz cell on a Shimadzu UV-2700 spectrophotometer equipped with a temperature controller.

### Fluorescence spectroscopy

Steady-state fluorescence spectra were recorded in a conventional quartz cell on a Hitachi F-4700 equipped with a temperature controller.

### TEM imaging

High-resolution transmission electron microscopy (TEM) images were acquired using a Talos F200X high-resolution transmission electron microscope operating at an accelerating voltage of 200 keV. The sample for high-resolution TEM measurements was prepared by dropping the solution onto a copper grid. The grid was then dried in vacuo under 420 nm irradiation for light-induced SP-PEI and thermal-relaxed SMC-SP-PEI self-assemblies at room temperature for immediate investigation of thermal-relaxation process.

### SEM imaging

Scanning electron microscopy (SEM) images were obtained using a FEI Inspect F50 scanning electron microscope. The sample for high-resolution SEM measurements was prepared by dropping the solution onto a silicon wafer. The wafer was then dried in vacuo under 420 nm irradiation for light-induced SP-PEI and thermal-relaxed SMC-SP-PEI self-assemblies at room temperature for immediate investigation of thermal-relaxation process.

### DLS spectroscopy

Solution samples were examined on a laser light scattering spectrometer (BI-200SM) equipped with a digital correlator (TurboCorr) at 636 nm at a scattering angle of 90°. The hydrodynamic diameter (Dh) was determined by DLS experiments at room temperature.

### Zeta potential

Solution samples were examined on a laser light scattering spectrometer (BI-200SM) equipped with Pt electrode at room temperature.

### NMR spectroscopy

^1^H-NMR spectra were recorded on a Bruker 600 MHz spectrometer. ^1^H-NMR measurements of SMC-PEI: 30 mL SMC (0.225 mM) and PEI (5 μg mL^–1^) aqueous solution was firstly prepared at 25 °C and kept in the dark. Then the solution was freeze-dried in the dark. After drying, the powder was dissolved in DMSO-d_6_ and measure the ^1^H-NMR_._^1^H-NMR measurements of SP-PEI: 30 ml SMC (0.225 mM) and PEI (5 μg mL^–1^) aqueous solution was firstly prepared at 25 °C and irradiated under 420 nm for 10 min. Then the solution was freeze-dried under 420 nm. After drying, the powder was dissolved in DMSO-d_6_ and then measured immediately by ^1^H-NMR. ^1^H-NMR measurements of SMC-SP-PEI: 30 ml SMC (0.225 mM) and PEI (5 μg mL^–1^) aqueous solution was firstly prepared at 25 °C and irradiated under 420 nm for 10 min and kept in the dark for 30 min. Then the solution was freeze-dried in the dark. After drying, the powder was dissolved in DMSO-d_6_ and measure the ^1^H-NMR.

### DLS measurements of thermal relaxation from SP-PEI to SMC-SP-PEI self-assemblies

Because the single measurement time of dynamic light scattering was much longer than the process of light-induced self-assembly of SP-PEI, the light-induced deformation process could not be investigated immediately from the DLS instrument. The thermal relaxation process from SP-PEI to SMC-SP-PEI was tested right after the irradiation under 420 nm light of the solution for 1 min. Then the variation of the particle size data was recorded on the screen by screen recording software from Windows10 system. We then recorded the DLS data every 5 s.

### UV-Vis measurements of reversibility between SP-PEI and SMC-SP-PEI self-assemblies

Spectrum and kinetics modes of UV-Vis were firstly used for the measurements. For the light-induced processes from SMC-SP-PEI to SP-PEI, the spectrum mode was used at the fastest scan speed (high scan speed, one point every 5 nm) in order to avoid the thermal relaxation of SP during the scanning. All the time of sampling and scanning was about 30 s. The kinetics mode was used for scanning the thermal dissociation processes. The 424 nm absorbance and 650 nm transmittance of the samples were measured soon (about 5 s) after irradiation for 1 min.

### pH measurements of reversibility between SP-PEI and SMC-SP-PEI solution

Before the 420 nm irradiation, the camera was focused at the display screen of the pH meter. Screen recording was immediately started upon the 420 nm irradiation. The pH variation during thermal relaxation process was recorded in the dark until the pH was not changed anymore. The data processing method is taking a point every 5 s from the pH in the video.

### Fluorescence measurements of reversibility between SP-PEI and SMC-SP-PEI solution

Wavelength scan and time scan modes were both used for fluorescence variation of dissipative assemblies. For the light-induced processes from SMC-SP-PEI to SP-PEI, the spectrum mode was used at the fastest scan speed (12,000 nm min^–1^) in order to avoid the thermal relaxation of SP during the scanning. All the time of sampling and scanning was about 10 s. The kinetics mode was used for scanning the thermal dissociation processes. The fluorescence of the samples was measured soon (about 5 s) after irradiation for 1 min.

### Observation of photodeformation between the SMC-SP-PEI and SP-PEI dissipative self-assemblies from laser scanning confocal microscope after loading fluorophores (SRB or CFDA)^48^

SRB and CFDA were employed respectively by loading in the DSAs for investigating the dissipative process between SMC-SP-PEI and SP-PEI nanoparticles. For SRB, fluorescence confocal images were collected of a solution containing SMC (0.225 mM), PEI (5 μg mL^–1^) in the presence of SRB (1 μM). The sample was under 420 nm irradiation for 10 min and then kept in the dark for 3 h. The dynamics of fluorescent nanoparticles emergence and disappearance were followed by collecting a time-series of images at the confocal microscope with temporal intervals of 5 s under 420 nm UV irradiation and 5 min in the dark. Fluorescence images were analyzed with ImageJ-Fiji software in order to count the particle numbers in each image and also to make a video from the time-series. The video of confocal images and the graph reporting the time evolution of the particle numbers were animated with Adobe Premiere Pro CC 2018 software. The time-series stack was analyzed using the selected parameters to extract the particle count from each image. The background threshold level was set to 10-255 (for 8-bit images) and the minimum object dimension was set to 3 pixel. Discarding the data set at the lowest threshold and size, for all analysis configurations, the amplitude of the highest peak was 12 times than the corresponding control mean value, confirming the relevance of such signature with respect to the background noise. For CFDA, Fluorescence confocal images were collected of a solution containing SMC (0.225 mM), PEI (5 μg mL^–1^) in the presence of CFDA (10 μM) and then treated as same as the SRB-loaded dissipative nanoparticles. Discarding the data set at the lowest threshold and size, for all analysis configurations, the amplitude of the highest peak was 26 times than the corresponding control mean value, confirming the relevance of such signature with respect to the background noise.

### Determination of the half-lives from the time-dependent variation during the thermal relaxation from SP-PEI to SMC-SP-PEI self-assemblies

The half-lives were determined by reading the time that the corresponding data (absorbance, transmittance, DLS data, pH, and fluorescence) reached the average of the initial and the end state in SP-PEI and SMC-SP-PEI self-assembly systems.

### Statistics and reproducibility

Each experiment was performed with three replicates. Each measurement was taken from three distinct samples. The results indicate mean ± standard deviation (SD).

## Supplementary information

Supplementary Information

Description of Additional Supplementary Files

Supplementary Movie 1

Supplementary Movie 2

Supplementary Movie 3

Supplementary Movie 4

Supplementary Movie 5

## Data Availability

The authors declare that data supporting the findings of this study are available within the paper and its Supplementary Information, and also from the authors upon request.
