# Supplementary material for: Light-activated photodeformable supramolecular dissipative self-assemblies
Source: Nat Commun. 2022 Jun 9;13:3216. doi: 10.1038/s41467-022-30969-2 (PMC9184535; doi:10.1038/s41467-022-30969-2)
Supplement: Supplementary file 1 — Supplementary Information [file 41467_2022_30969_MOESM1_ESM.pdf]

Supplementary Information

Light-activated photodeformable supramolecular dissipative self-assemblies

Xu-Man Chen,[1] Wei-Jie Feng,[1] Hari Krishna Bisoyi,[2] Shu Zhang,[1] Xiao Chen,[1] Hong Yang*[1] and Quan Li*[1, 2]

[1]Institute of Advanced Materials, School of Chemistry and Chemical Engineering, and Jiangsu Province Hi-Tech Key Laboratory for Biomedical Research, Southeast University, Nanjing 211189, China; [2]Advanced Materials and Liquid Crystal Institute and Chemical Physics Interdisciplinary Program, Kent State University, Kent, OH 44242, USA

Email: quanli3273@gmail.com; yangh@seu.edu.cn;

Table of Contents

A. Materials and methods

B. Construction of light-activated photodeformable supramolecular dissipative self-assemblies

C. Dissipative properties of the photodeformable supramolecular dissipative self-assemblies

D. Dynamic control of fluorophores with different colors

E. Creating self-erasing multicolor fluorescent images

F. ^{1}H-NMR, ^{13}C-NMR and HRMS spectra

Section A. Materials and methods

Materials

Chemical reagents were purchased from commercial resource unless noted. 2,3,3-Trimethylindolenine (purity: 99%) was purchased from Aladdin Reagent (Shanghai) Co., Ltd. propane sultone (purity: 98%), 2-hydroxybenzaldehyde (purity: 98%), Polyethyleneimine (M_w = 10000) (purity: 99%) were purchased from Energy chemical Co., Ltd. Ethanol (purity: 99.5%) and diethyl ether (purity: 99.7%) were purchased from Sinopharm Chemical Reagent Co., Ltd. CFDA (purity: 90%) and Rh101 (purity: 98%) were purchased from Shanghai Macklin Biochemical Co., Ltd. Scoparone (purity: 98%) and SRB (purity: 95%) were purchased from Shanghai yuanye Bio-Technology Co., Ltd.

Methods

Synthesis of SMC. SMC was synthesized according to published literature[1], 2,3,3-trimethylindolenine (1.65 g, 0.01 mmol) was firstly added into propane sultone (1.26 g, 0.01 mmol). The mixture was stirred at 90 °C for 4 h under N_2. The purple solid was collected by filtration, washed with cold diethyl ether, and dried in vacuo to generate 2,3,3-trimethyl-1-(3-sulfonatepropyl)-3H-indolium. Then, 2,3,3-trimethyl-1-(3-sulfonatepropyl)-3H indolium (100 mg, 0.36 mmol) and 2-hydroxybenzaldehyde (48 mg, 0.39 mmol) were added into anhydrous ethanol (2 mL). The mixture was allowed to reflux overnight. The orange solid SMC was obtained by filtration. The structure was determined by ^{1}H-NMR, ^{13}C-NMR and HRMS. ^{1}H-NMR (600 MHz, DMSO-d_6): δ 11.04 (s, 1H), 8.60 (d, J = 16.4 Hz, 1H), 8.29 (d, J = 7.3 Hz, 1H), 8.03 (d, J = 7.6 Hz, 1H), 7.88 (t, J = 11.9 Hz, 2H), 7.69 – 7.54 (m, 2H), 7.53 – 7.43 (m, 1H), 7.04 (d, J = 8.1 Hz, 1H), 6.99 (t, J = 7.5 Hz, 1H), 4.88 – 4.69 (m, 3H), 4.36 – 4.00 (m, 1H), 2.77 – 2.57 (m, 3H), 2.35 – 2.08 (m, 3H), 1.77 (s, 7H).^{13}C-NMR (151 MHz, DMSO-d_6): δ 181.16, 158.37, 148.05, 142.82, 140.28, 135.08, 129.11, 128.54, 128.52, 122.32, 120.73, 119.40, 116.00, 114.44, 110.79, 51.08, 46.84, 44.26, 25.43, 23.39. ESI-MS: m/z calculated for: $C_{21}H_{23}NO_4S$: requires 386.14206 for $[M+H]^+$, found 386.14424.

The preparation for SP-PEI and SMC-SP-PEI photodeformable dissipative self-

assemblies. SMC (0.225 mM) and PEI (5 μg mL^{-1}) aqueous solution was firstly prepared at 25 °C. Then the solution was exposed under 420 nm light (15 mW cm^{-2} unless mentioned) for 10 min for light-induced assembly, SP-PEI, for further measurements. Then, SP-PEI self-assemblies was placed in dark for ~1600 s and the self-assemblies gradually transformed to SMC-SP-PEI assemblies.

NMR spectroscopy. NMR spectra were recorded on a Bruker 600 MHz spectrometer.

ESI-MS spectroscopy. Electrospray ionization mass spectra (ESI-MS) were measured by Agilent 1260-6224.

UV-Vis spectroscopy. UV-Vis spectra and the optical transmittance were recorded in a quartz cell on a Shimadzu UV-2700 spectrophotometer equipped with a temperature controller.

Fluorescence spectroscopy. Steady-state fluorescence spectra were recorded in a conventional quartz cell on a Hitachi F-4700 equipped with a temperature controller.

TEM imaging. High-resolution Transmission electron microscopy (TEM) images were acquired using a Talos F200X high-resolution transmission electron microscope operating at an accelerating voltage of 200 keV. The sample for high-resolution TEM measurements was prepared by dropping the solution onto a copper grid. The grid was then air-dried under 420 nm irradiation for light-induced SP-PEI and thermal-relaxed SMC-SP-PEI self-assemblies at room temperature.

SEM imaging. Scanning electron microscopy (SEM) images were obtained using a FEI Inspect F50 scanning electron microscope. The sample for high-resolution SEM measurements was prepared by dropping the solution onto a silicon wafer. The wafer was then air-dried under 420 nm irradiation for light-induced SP-PEI and thermal-relaxed SMC-SP-PEI self-assemblies at room temperature.

DLS spectroscopy. Solution samples were examined on a laser light scattering spectrometer (BI-200SM) equipped with a digital correlator (TurboCorr) at 636 nm at a scattering angle of 90°. The hydrodynamic diameter (Dh) was determined by DLS experiments at room temperature.

Zeta potential. Solution samples were examined on a laser light scattering

spectrometer (BI-200SM) equipped with Pt electrode at room temperature.

DLS measurements of thermal relaxation from SP-PEI to SMC-SP-PEI self-assemblies. Because the single measurement time of dynamic light scattering is much longer than the process of light-induced self-assembly of SP-PEI, the light-induced deformation process could not be investigated immediately from the DLS instrument. The thermal relaxation process from SP-PEI to SMC-SP-PEI was tested right after the irradiation under 420 nm light of the solution for 1 min. Then the variation of the particle size data was recorded on the screen by screen recording software from Windows10 system. We then recorded the DLS data every 5 s.

UV-Vis measurements of reversibility between SP-PEI and SMC-SP-PEI self-assemblies. Spectrum and kinetics modes of UV-Vis were firstly used for the measurements. For the light-induced processes from SMC-SP-PEI to SP-PEI, the spectrum mode was used at the fastest scan speed (high scan speed, one point every 5 nm) in order to avoid the thermal relaxation of SP during the scanning. All the time of sampling and scanning was about 30 s. The kinetics mode was used for scanning the thermal dissociation processes. The 424 nm absorbance and 650 nm transmittance of the samples were measured soon (about 5 s) after irradiation for 1 min.

pH measurements of reversibility between SP-PEI and SMC-SP-PEI solution. Before the 420 nm irradiation, the camera was focused at the display screen of the pH meter. Screen recording was immediately started upon the 420 nm irradiation. The pH variation during thermal relaxation process was recorded in the dark until the pH was not change any more. The data processing method is taking a point every five seconds from the pH in the video.

Fluorescence measurements of reversibility between SP-PEI and SMC-SP-PEI solution. Wavelength scan and time scan modes were both used for fluorescence variation of dissipative assemblies. For the light-induced processes from SMC-SP-PEI to SP-PEI, the spectrum mode was used at the fastest scan speed (12000 nm min^{-1}) in order to avoid the thermal relaxation of SP during the scanning. All the time of sampling and scanning was about 10 s. The kinetics mode was used for scanning the thermal

dissociation processes. The fluorescence of the samples was measured soon (about 5 s) after irradiation for 1 min.

Observation of photodeformation between the SMC-SP-PEI and SP-PEI dissipative self-assemblies from laser scanning confocal microscope after loading fluorophores (SRB or CFDA).[1] SRB and CFDA were employed respectively by loading in the DSAs for investigating the dissipative process between SMC-SP-PEI and SP-PEI nanoparticles. For SRB, fluorescence confocal images were collected of a solution containing SMC (0.225 mM), PEI (5 μg mL^{-1}) in the presence of SRB (1 μM). The sample was under 420 nm irradiation for 10 min and then kept in the dark for 3 h. The dynamics of fluorescent nanoparticles emergence and disappearance was followed by collecting a time-series of images at the confocal microscope with temporal intervals of 5 s under 420 nm UV irradiation and 5 min in the dark. Fluorescence images were analyzed with ImageJ-Fiji software in order to count the particle numbers in each image and also to make a video from the time-series. The video of confocal images and the graph reporting the time evolution of the particle numbers were animated with Adobe Premiere Pro CC 2018 software. The time-series stack has been analyzed using the selected parameters to extract the particle count from each image. The background threshold level has been set to 10-255(for 8-bit images) and the minimum object dimension has been set to 3 pixel. Discarding the data set at the lowest threshold and size, for all analysis configurations, the amplitude of highest peak in is 12 times than the corresponding control mean value, confirming the relevance of such signature with respect to the background noise. For CFDA, Fluorescence confocal images were collected of a solution containing SMC (0.225 mM), PEI (5 μg mL^{-1}) in the presence of CFDA (10 μM) and then treated as same as the SRB-loaded dissipative nanoparticles. Discarding the data set at the lowest threshold and size, for all analysis configurations, the amplitude of highest peak in is 26 times than the corresponding control mean value, confirming the relevance of such signature with respect to the background noise.

Determination of the half-lives from the time-dependent variation during the thermal relaxation from SP-PEI to SMC-SP-PEI self-assemblies. The half-lives

were determined by reading the time that the corresponding data (absorbance, transmittance, DLS data, pH and fluorescence) reached the average of the initial and the end state in SP-PEI and SMC-SP-PEI self-assembly systems.

Supplementary Movies. Supplementary Movie 1 and 2 showed the confocal images of light-induced deformation from SMC-SP-PEI and SP-PEI self-assemblies and its thermal relaxation back to SMC-SP-PEI in the dark by loading SRB and CFDA as well as total number of fluorescent particle counts detected over time. Supplementary Movie 3, 4 and 5 showed dynamic fluorescent variation of loaded scoparone, CFDA and Rh101 over time during light-induced deformation and thermal relaxation process, respectively.

Section B. Construction of light-activated photodeformable supramolecular dissipative self-assemblies[1]

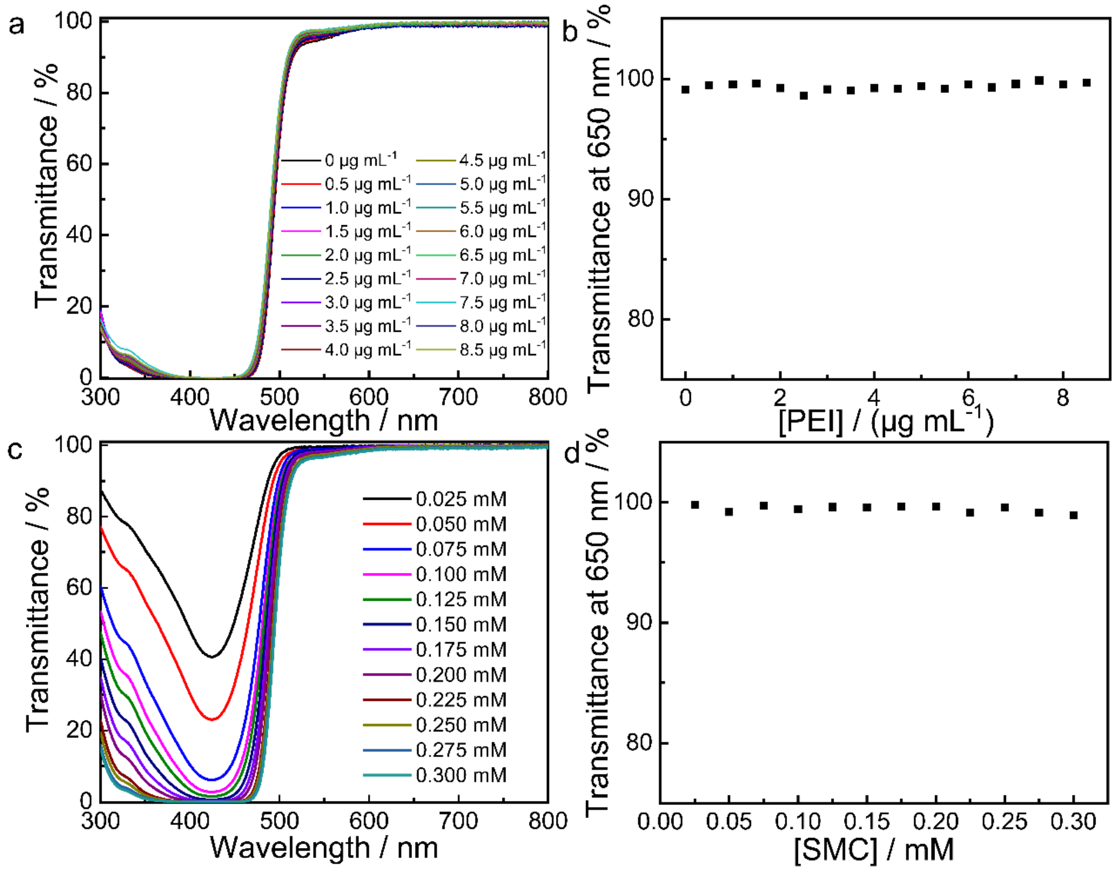

Supplementary Figure 1. (a) Dependence of transmittance of SMC (0.225 mM) in varying concentration of PEI (0 – 8.5 μg mL^{-1}) without irradiation. (b) Transmittance variation at 650 nm of a). (c) Dependence of transmittance of PEI (5 μg mL^{-1}) in varying

concentration of SMC (0.025 mM – 0.3 mM) without irradiation. (d) Variation of transmittance at 650 nm of (c).

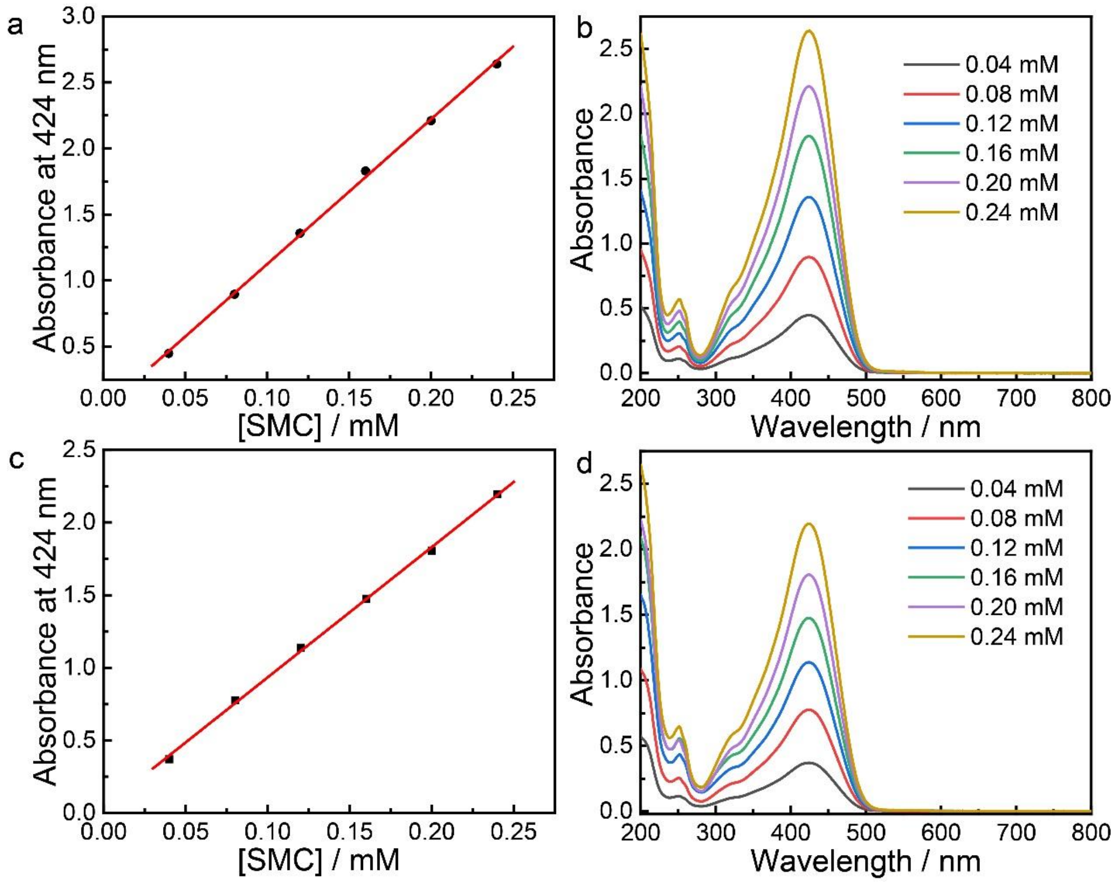

Supplementary Figure 2. (a) Standard curves of absorbance at 424 nm of SMC on increasing in concentration (0.04 mM – 0.24 mM) in aqueous solution. The calibration equation in **a** is: $A(424\ \ nm) = 0.026 + 10.982 \times c(SMC), R^2 = 0.999.$ (b) UV-Vis absorbance spectra of (a). (c) Standard curves of absorbance at 424 nm of SMC-PEI on increasing in concentration (0.04 mM – 0.24 mM) in aqueous solution (PEI fixed at 5 μg mL^{-1}). The calibration equation in **a** is: $A(424\ \ nm) = 0.039 + 8.965 \times c(SMC), R^2 = 0.999.$ (d) UV-Vis absorbance spectra of (c).

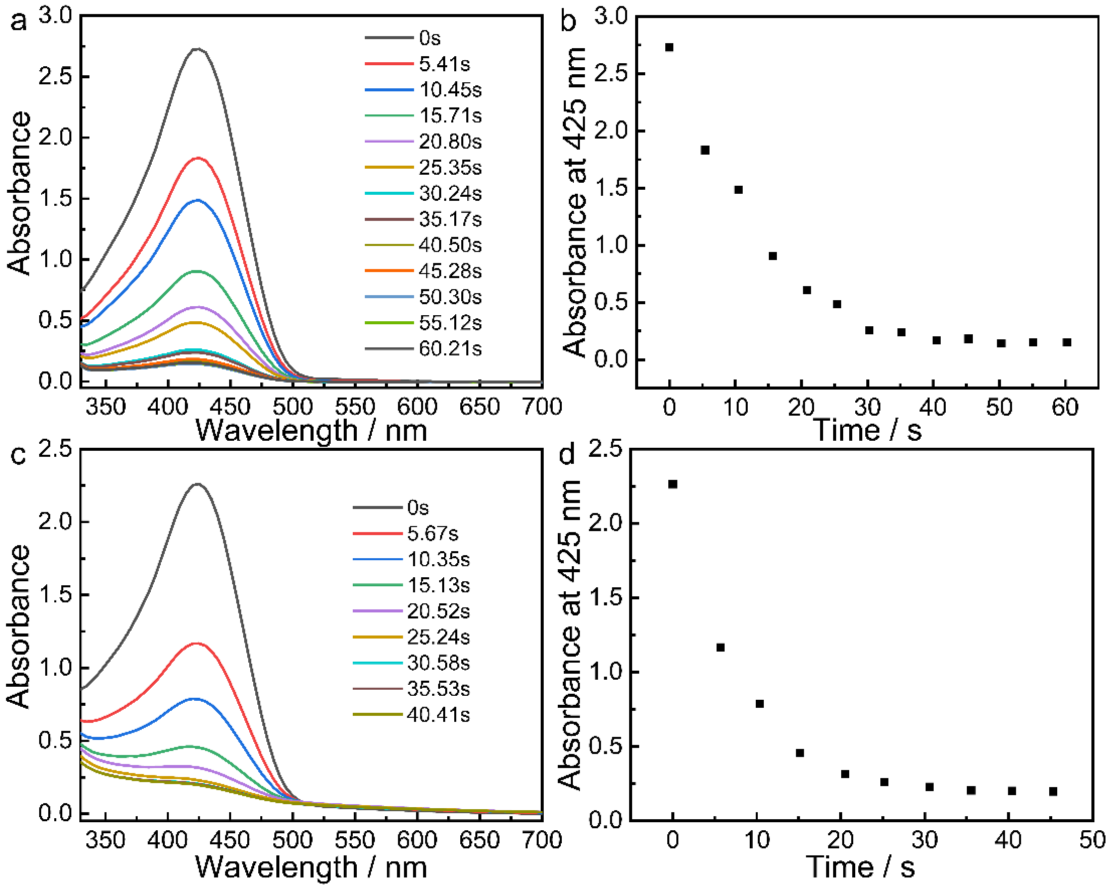

Supplementary Figure 3. (a) UV-Vis absorbance spectra of SMC (0.225 mM) in aqueous solution upon irradiation with different time length (0 s – 65.21 s). (b) Absorbance at 425 nm of spectrum (a). (c) UV-Vis absorbance spectra of SMC-PEI (0.225 mM – 5 μg mL^{-1}) in aqueous solution upon irradiation with different time length (0 s – 40.41 s). (d) Absorbance at 425 nm of spectrum (c).

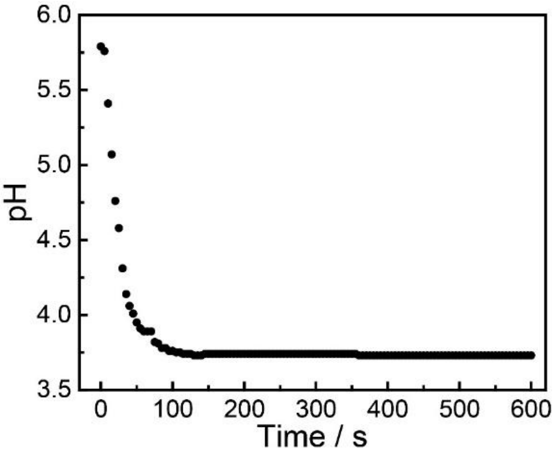

Supplementary Figure 4. pH decreasing process from SMC-PEI to SP-PEI upon irradiation for 600s. ($[SMC]_{initial}$ = 0.225 mM, [PEI] = 5 μg mL^{-1}).

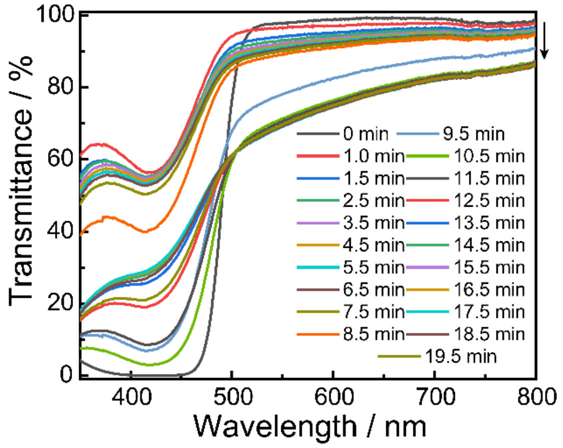

Supplementary Figure 5. Variation of transmittance spectra of SMC-PEI (0.225 mM – 5 μg mL^{-1}) solution upon irradiation for different time length (0 min – 19.5 min).

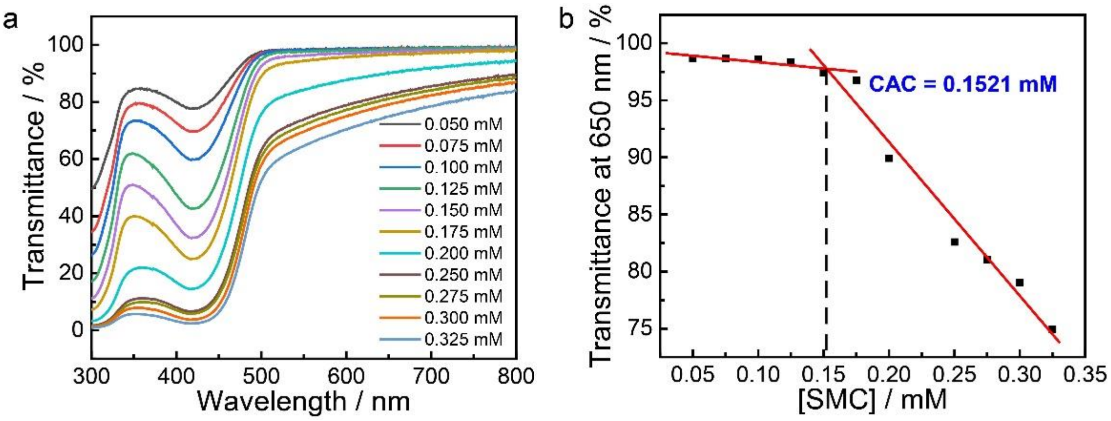

Supplementary Figure 6. (a) Transmittance of PEI (5 μg mL^{-1}) in varying concentration of SMC from 0.050 mM to 0.325 mM right after irradiation with 420 nm light for 15 min. (b) Transmittance at 650 nm of spectrum (a) and the CAC fitting graph.

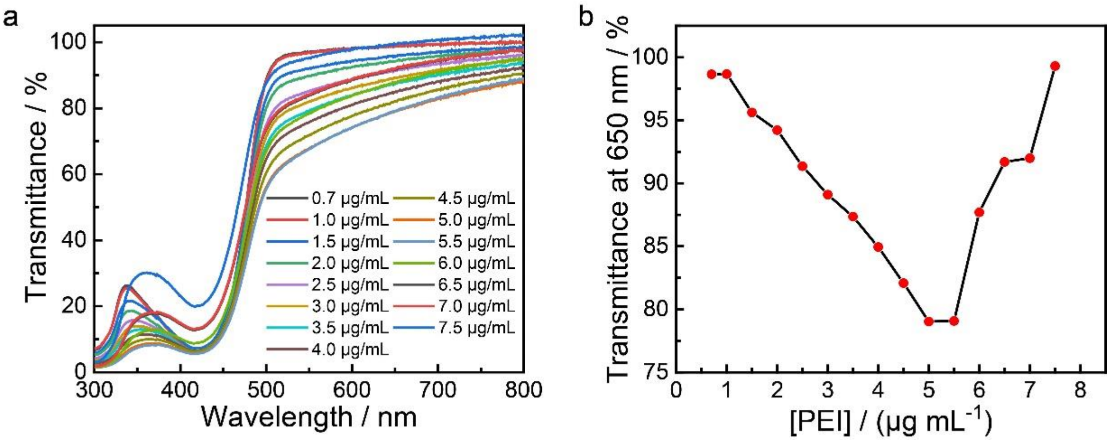

Supplementary Figure 7. (a) Transmittance of SMC (0.225 mM) in varying concentration of PEI (0.7 μg mL^{-1} – 7.5 μg mL^{-1}) right after 420 nm irradiation for 15

min. (b) Transmittance at 650 nm of spectrum (a).

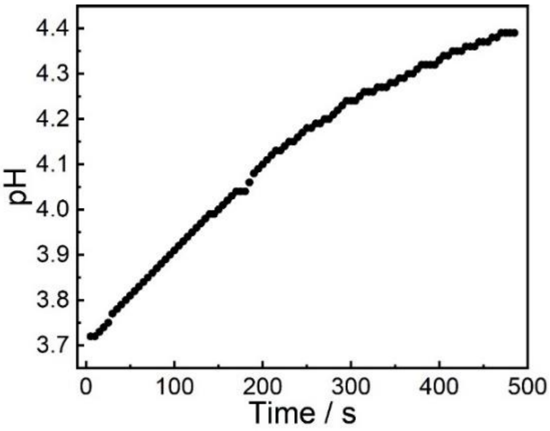

Supplementary Figure 8. pH increasing process of SMC-PEI in the dark in aqueous solution right after irradiation. ($[SMC]_{initial}$ = 0.225 mM, [PEI] = 5 μg mL^{-1})

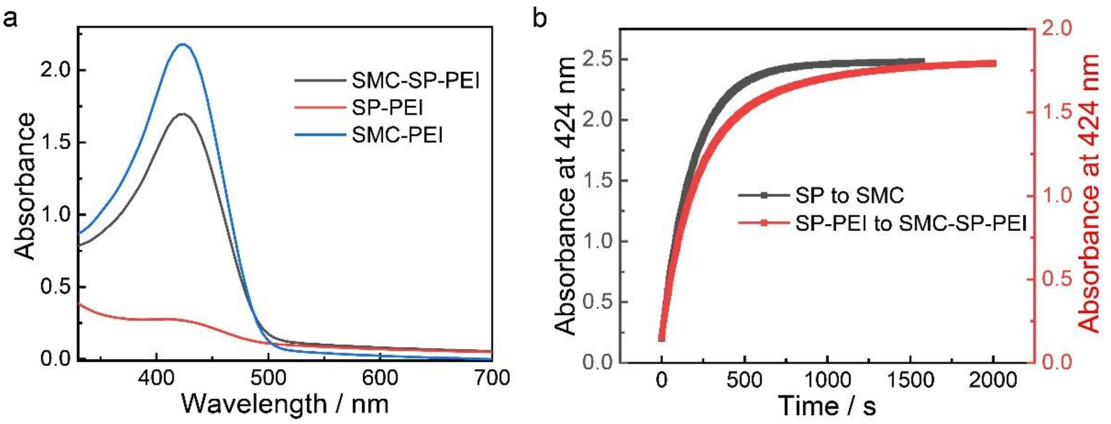

Supplementary Figure 9. (a) UV-Vis absorbance spectra of SMC-PEI, SP-PEI and SMC-SP-PEI (b) Kinetic process comparison of transmittance at 650 nm of the recovery process between SP-PEI and SMC-SP-PEI. ($[SMC]_{initial}$ = 0.225 mM, [PEI] = 5 μg mL^{-1})

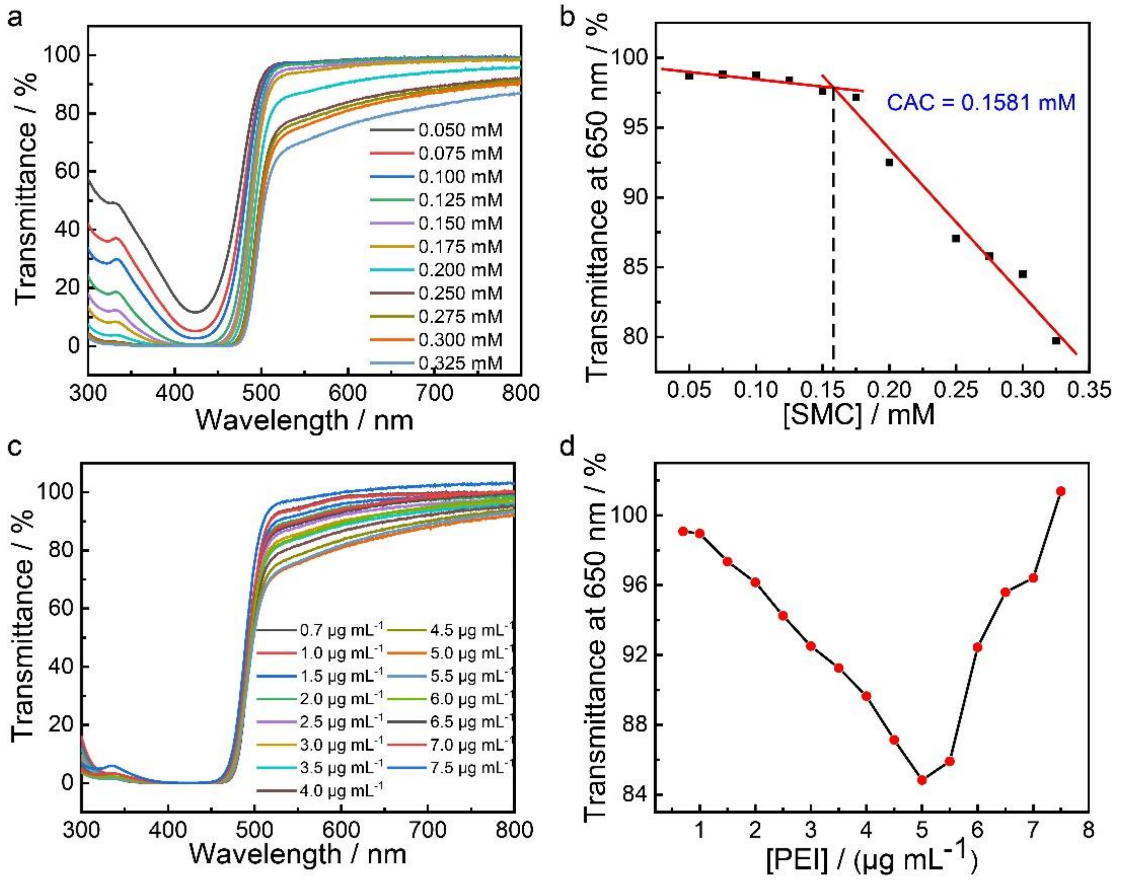

Supplementary Figure 10. (a) UV-Vis transmittance spectra of SMC-SP-PEI in varying concentration of SMC from 0.05 mM to 0.325 mM after 30 min recovery from SP-PEI in dark. (b) Transmittance at 650 nm of spectrum (a) and the CAC fitting graph. (c) UV-Vis transmittance spectra of SMC-SP-PEI in varying concentration of PEI from 0.7 mM to 7.5 mM after 30 min recovery from SP-PEI in dark. (d) Transmittance at 650 nm of spectrum (c).

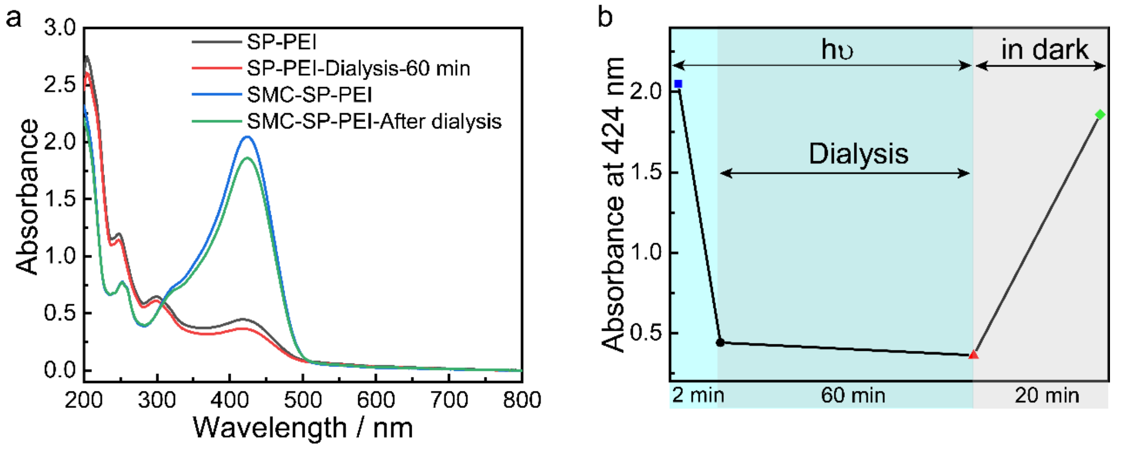

Supplementary Figure 11. (a) UV-Vis absorption of SMC-SP-PEI, SP-PEI, SP-PEI

right after dialysis for 1h, SMC-SP-PEI from the dialyzed SP-PEI in dark. (b) absorbance at 424 nm of (a) towards the time during the irradiation, dialysis and keeping in dark.

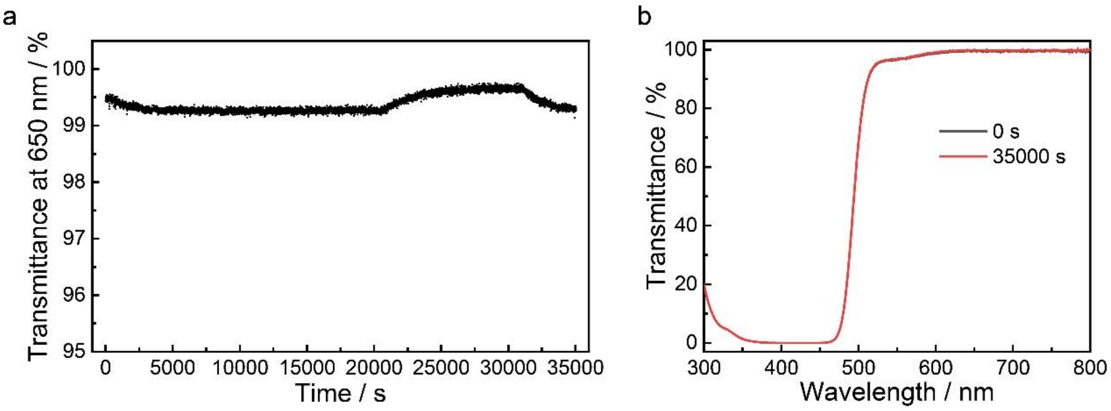

Supplementary Figure 12. (a) The transmittance at 650 nm of SMC-PEI kept in dark without irradiation for 35000 s. (b) UV-Vis transmittance spectra of (a) at 0 s and 35000 s. ($[SMC]_{initial}$ = 0.225 mM, [PEI] = 5 μg mL^{-1})

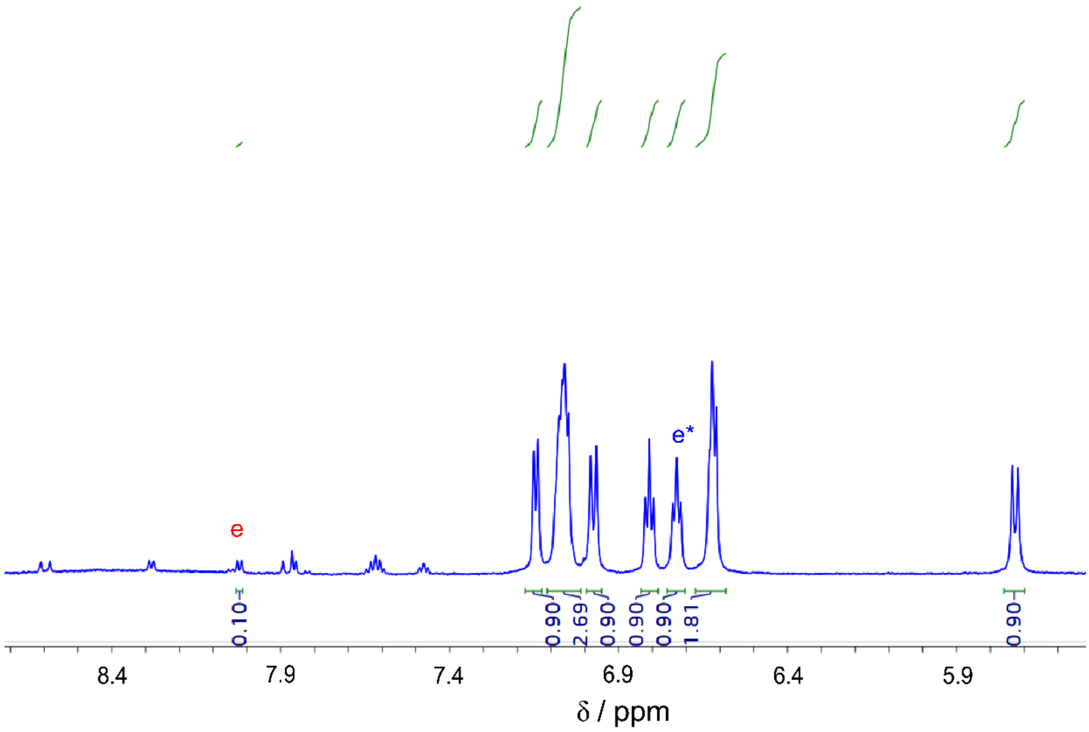

Supplementary Figure 13. Partial ^{1}H-NMR spectra (400 MHz, DMSO-d_6) of SP-PEI.

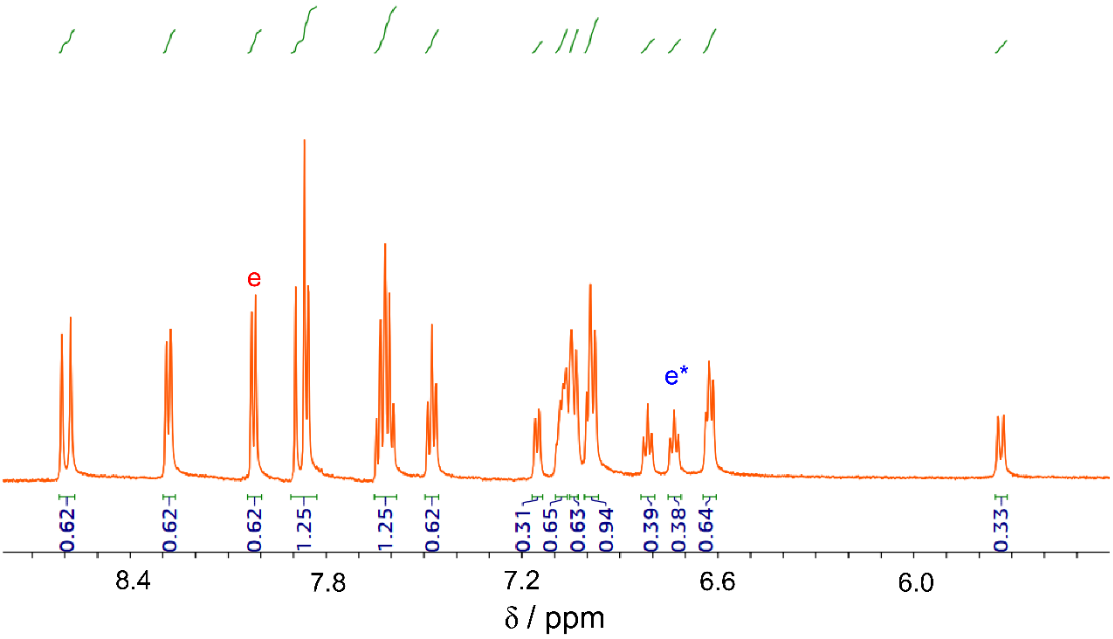

Supplementary Figure 14. Partial ^{1}H-NMR spectra (400 MHz, DMSO-d_6) of SMC-SP-PEI.

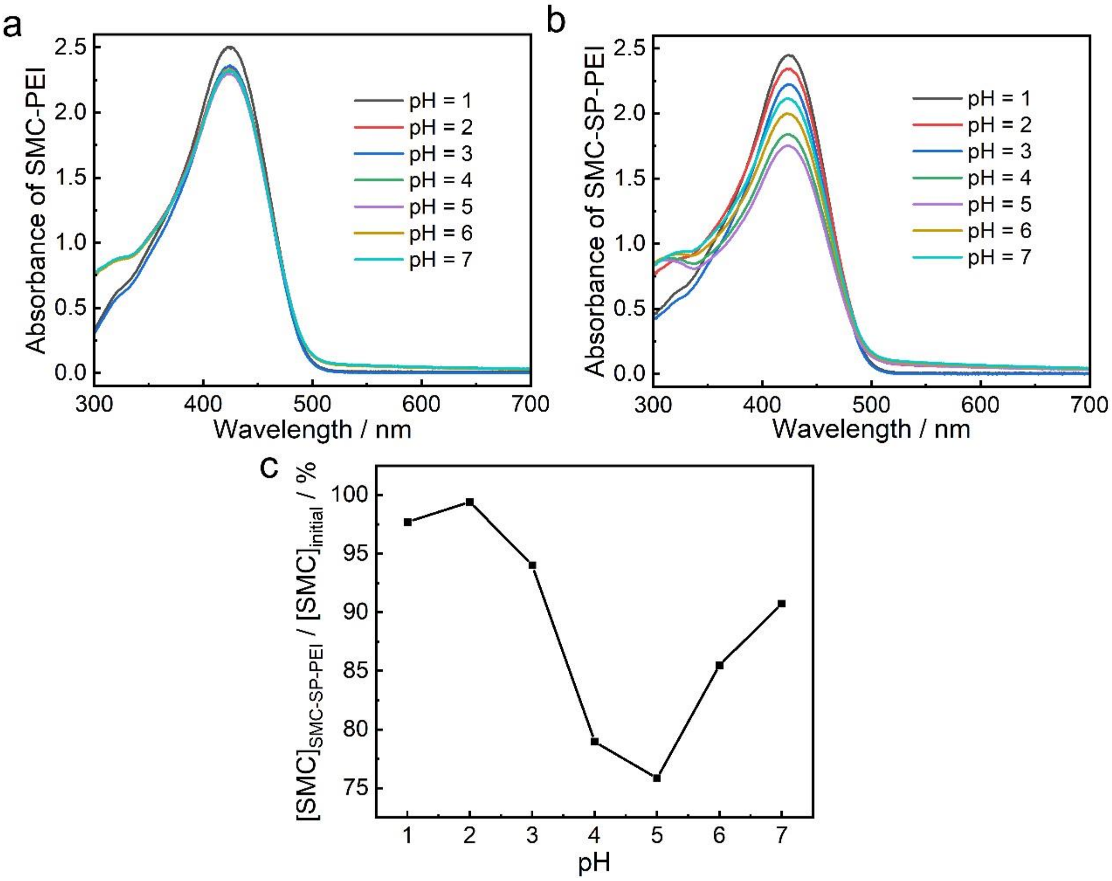

Supplementary Figure 15. (a, b) UV-Vis absorbance spectra of SMC-PEI and SMC-SP-PEI in varying pH (pH = 1–7), respectively. (c) SMC in SMC-SP-PEI state as a

percentage of initial state in varying pH (pH = 1–7) based on (a, b). ($[SMC]_{initial}$ = 0.225 mM, [PEI] = 5 μg mL^{-1})

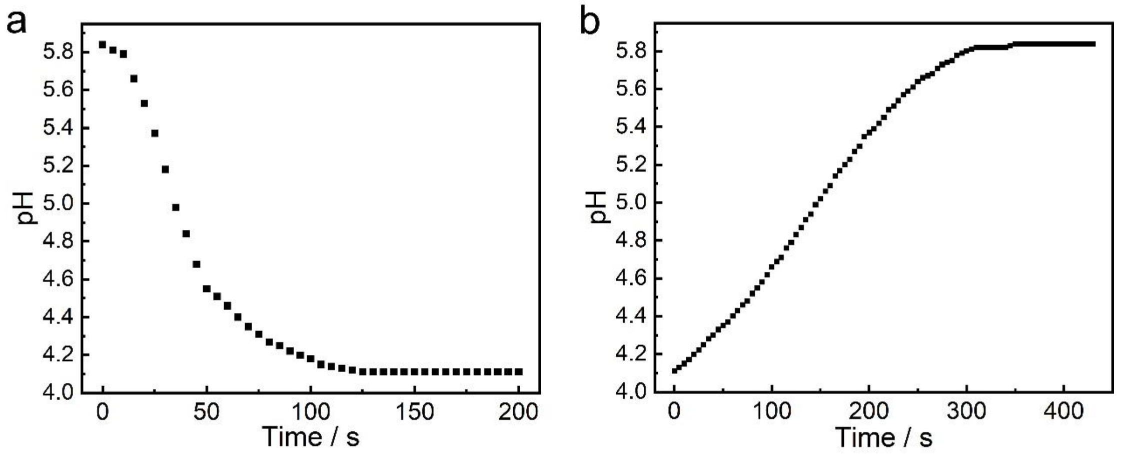

Supplementary Figure 16. (a) pH decreasing process from SMC to SP upon irradiation. (b) pH increasing process of SMC in the dark in aqueous solution right after irradiation. ($[SMC]_{initial}$ = 0.225 mM).

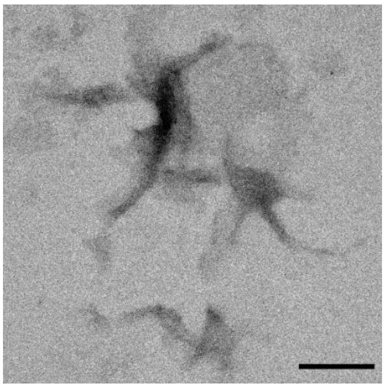

Supplementary Figure 17. TEM image of SMC-PEI without irradiation and the scale bar is 500 nm.

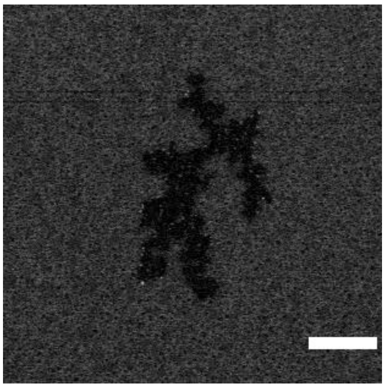

Supplementary Figure 18. SEM image of SMC-PEI without irradiation and the scale bar is 5 μm.

Section C. Dissipative properties of the photodeformable supramolecular dissipative self-assemblies

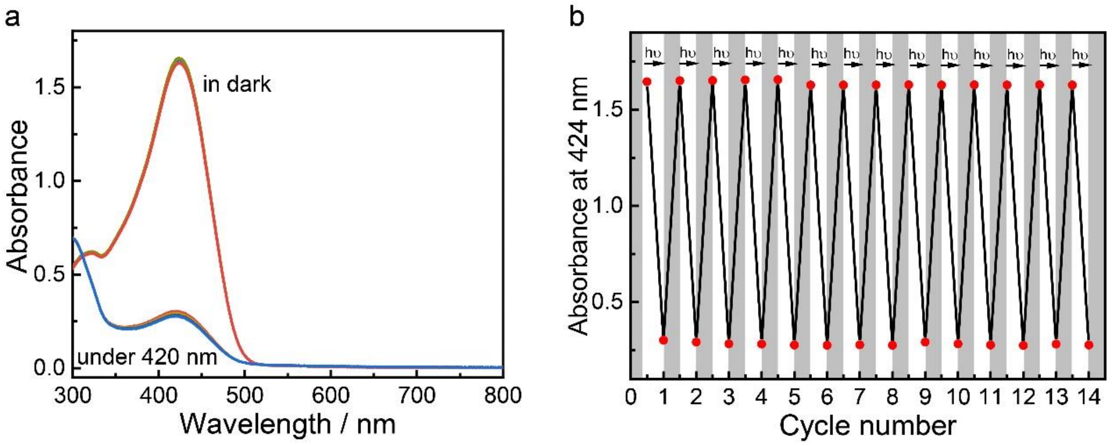

Supplementary Figure 19. (a) Absorption changing cycles of SMC-SP-PEI in aqueous solution after 130 s of irradiation under 420 nm light and kept in dark for 30 min at 25 °C. (b) changing cycles of absorbance at 424 nm of (a). ([SMC]$_{initial}$ = 0.225 mM, [PEI] = 5 μg mL^{-1})

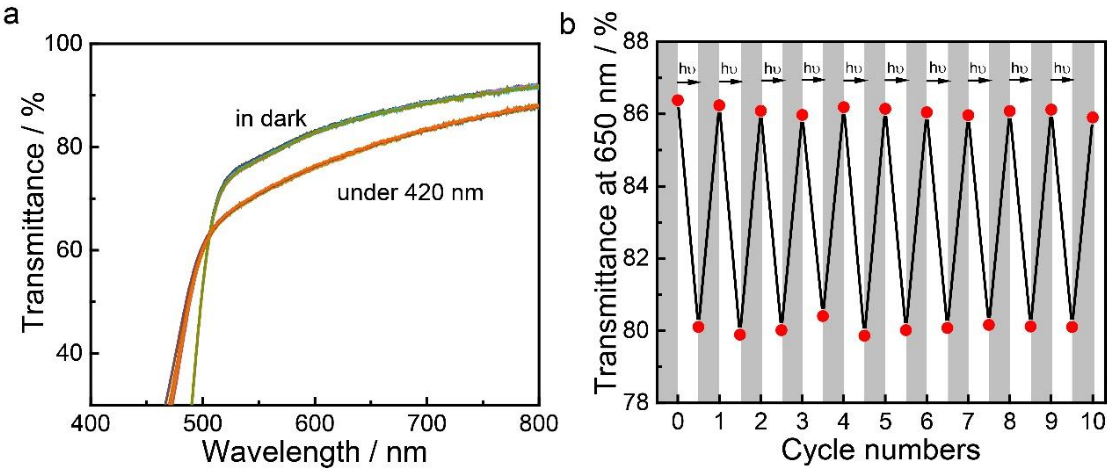

Supplementary Figure 20. (a) Transmittance changing cycles of SMC-SP-PEI in aqueous solution after 2 min of irradiation under 420 nm light and kept in dark for 30 min at 25 °C. (b) changing cycles of transmittance at 650 nm of (a). ([SMC]$_{initial}$ = 0.225 mM, [PEI] = 5 μg mL^{-1})

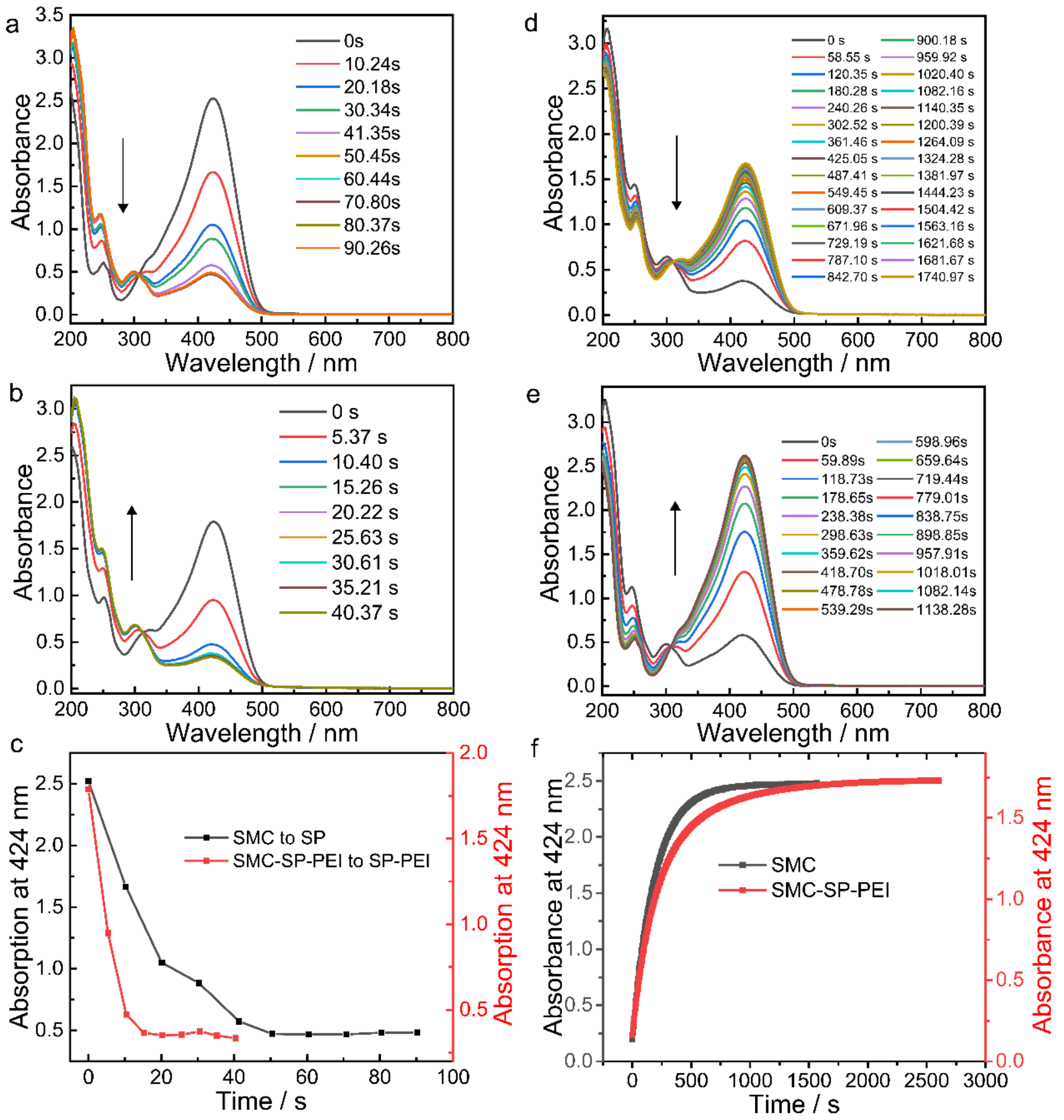

Supplementary Figure 21. (a-b) UV-Vis absorbance spectra of SMC and SMC-SP-PEI in aqueous solution upon irradiation with different time length (0 s – 100.31 s) and (0 s – 40.37 s). (c) Comparison of absorbance at 424 nm decreasing process during irradiation between SMC and SMC-PEI alone in aqueous solution of (a) and (b). (d-e) UV-Vis absorbance spectra of SMC and SMC-SP-PEI in aqueous solution after 130 s of irradiation and kept in dark at 25 °C with different time length (0 s – 1138.28 s) and (0 s – 1740.97 s). (f) Comparison of absorbance at 424 nm increasing kinetics after 130 s of irradiation and kept in dark at 25 °C between SMC and SMC-PEI alone in aqueous solution of (d) and (e). ($[SMC]_{initial}$ = 0.225 mM, [PEI] = 5 μg mL^{-1})

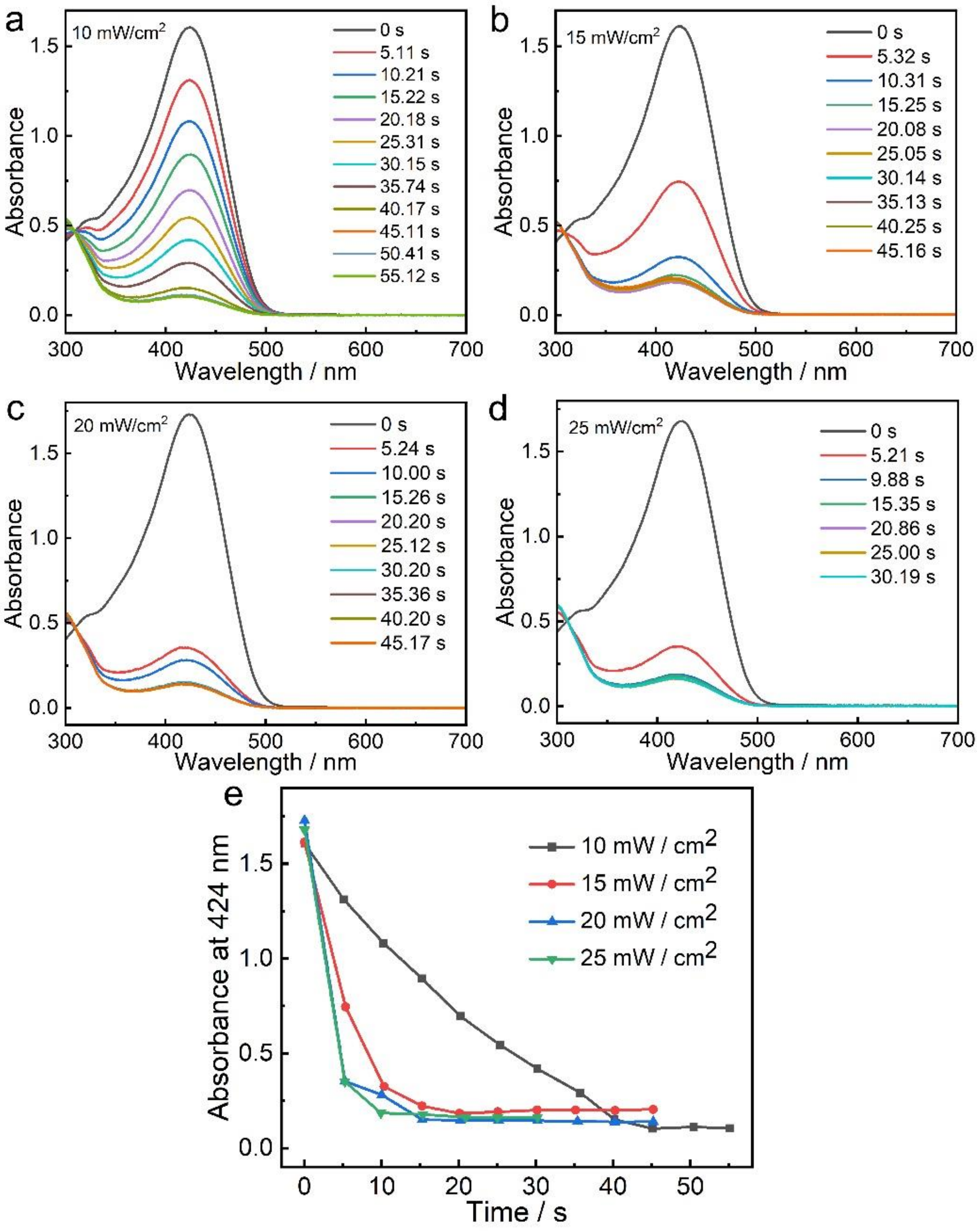

Supplementary Figure 22. (a-d) UV-Vis absorbance of SMC-PEI upon irradiation by 420 nm light (temperature: 25 °C) with different time length at different optical power density (10 mW/cm^2, 15 mW/cm^2, 20 mW/cm^2, 25 mW/cm^2, respectively). (e) Decreasing process of UV-Vis absorbance at 424 nm of (a-d). ([SMC]$_{initial}$ = 0.225 mM, [PEI] = 5 μg mL^{-1})

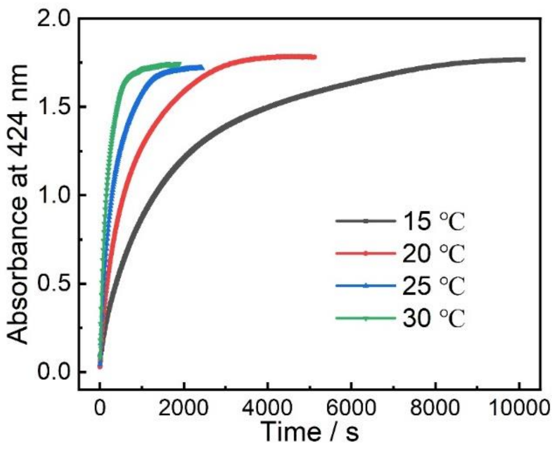

Supplementary Figure 23. Comparison of increasing kinetics of absorbance at 424 nm from SP-PEI to SMC-SP-PEI in the dark at different temperature (15 °C, 20 °C, 25 °C, 30 °C, respectively). ($[SMC]_{initial}$ = 0.225 mM, [PEI] = 5 μg mL^{-1})

Section D. Dynamic control of fluorophores with different colors

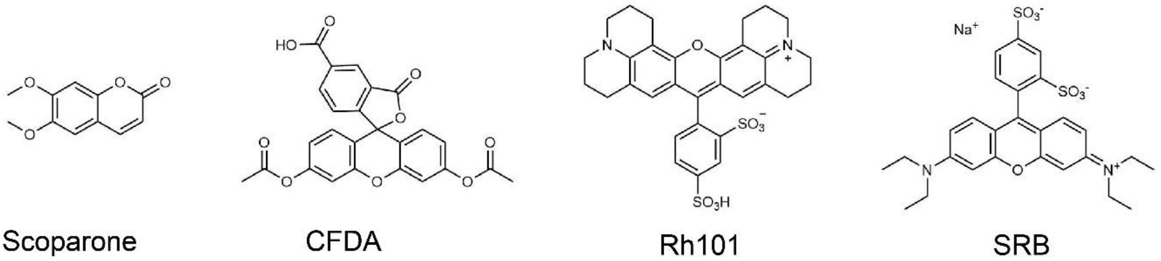

Supplementary Figure 24. Chemical structure of scoparone, CFDA, Rh101, SRB.

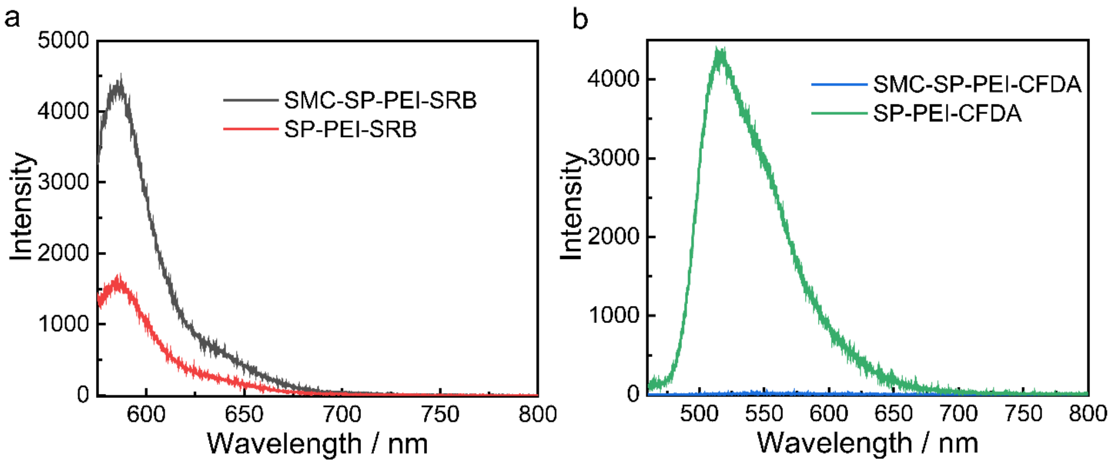

Supplementary Figure 25. (a) Fluorescence intensity of SMC-SP-PEI-SRB before and after irradiation by 420 nm light in aqueous solution. (b) Fluorescence intensity of SMC-SP-PEI-CFDA before and after irradiation by 420 nm light in aqueous solution. ($[SMC]_{initial}$ = 0.225 mM, [PEI] = 5 μg mL^{-1}, [SRB] = 0.001 mM, [CFDA] = 0.01 mM)

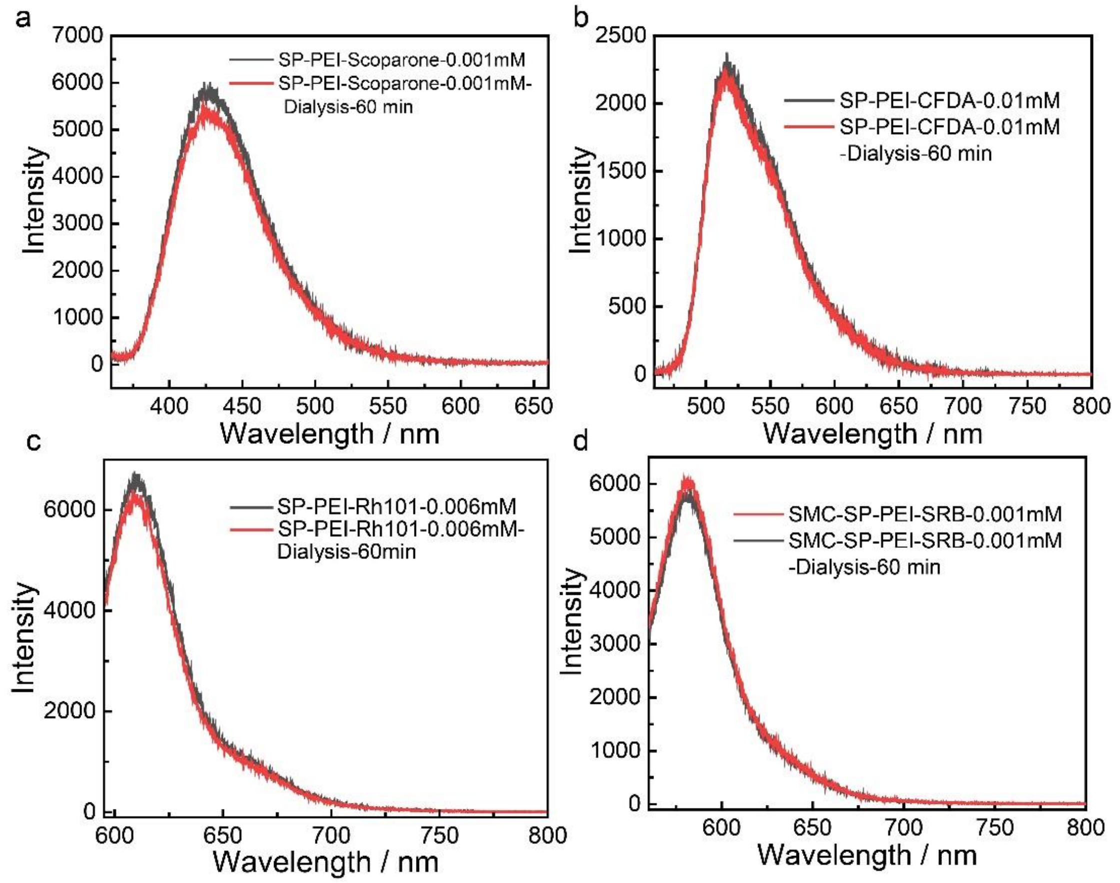

Supplementary Figure 26. (a-c) Fluorescence intensity of SMC-PEI loading dyes (scoparone, CFDA and Rh101) and dialyzed for 60 min under 420 nm irradiation. (d) Fluorescence intensity of SMC-PEI loading SRB and dialyzed for 60 min. ($[SMC]_{initial}$ = 0.225 mM, [PEI] = 5 μg mL^{-1}, [Scoparone] = 0.001 mM, [CFDA] = 0.01 mM, [Rh101] = 0.006 mM, [SRB] = 0.001 mM)

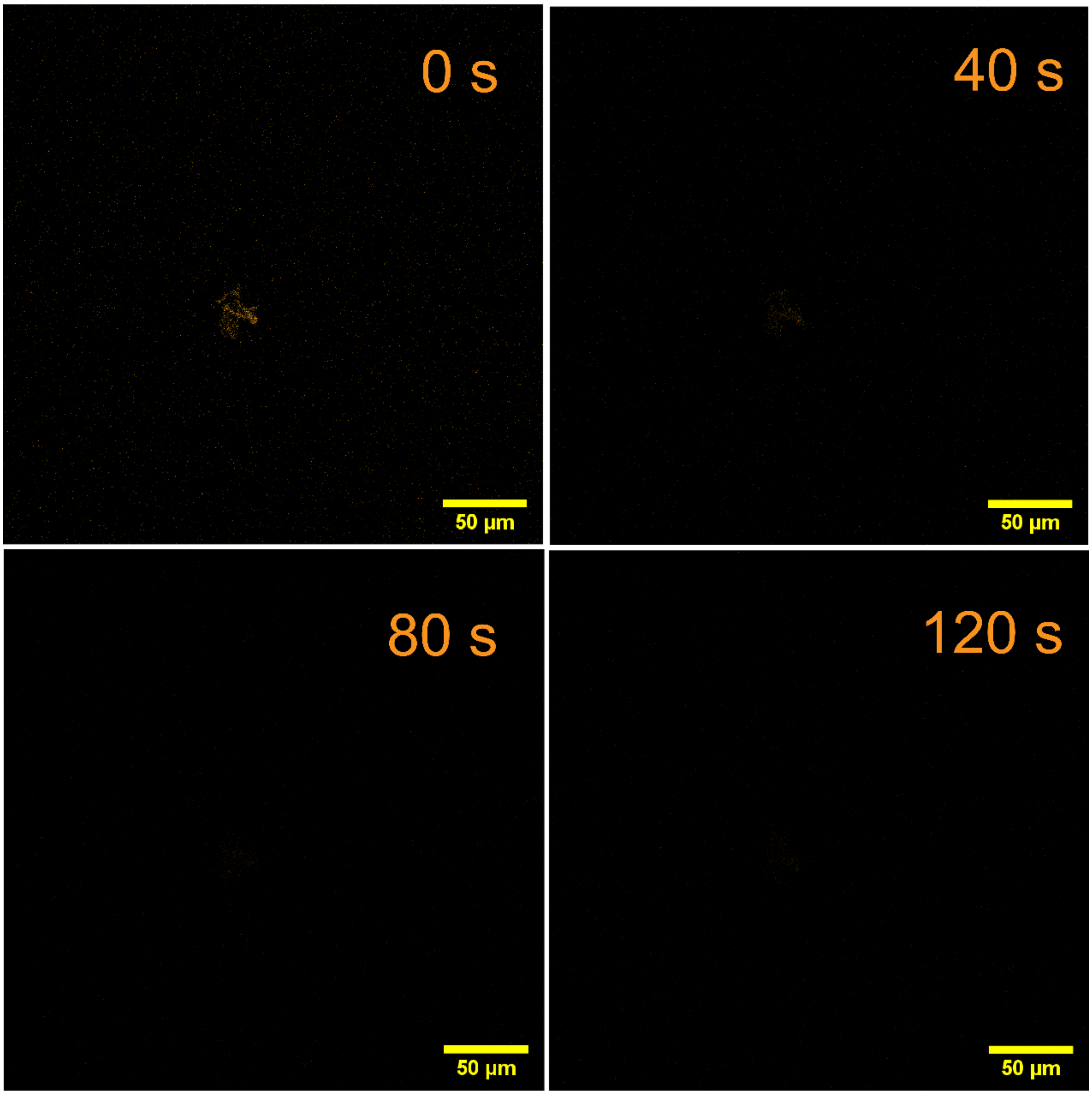

Supplementary Figure 27. Confocal images of SMC-SP-PEI-SRB irradiation process in aqueous solution over time. ($[SMC]_{initial}$ = 0.225 mM, [PEI] = 5 µg mL^{-1}, [SRB] = 0.001 mM)

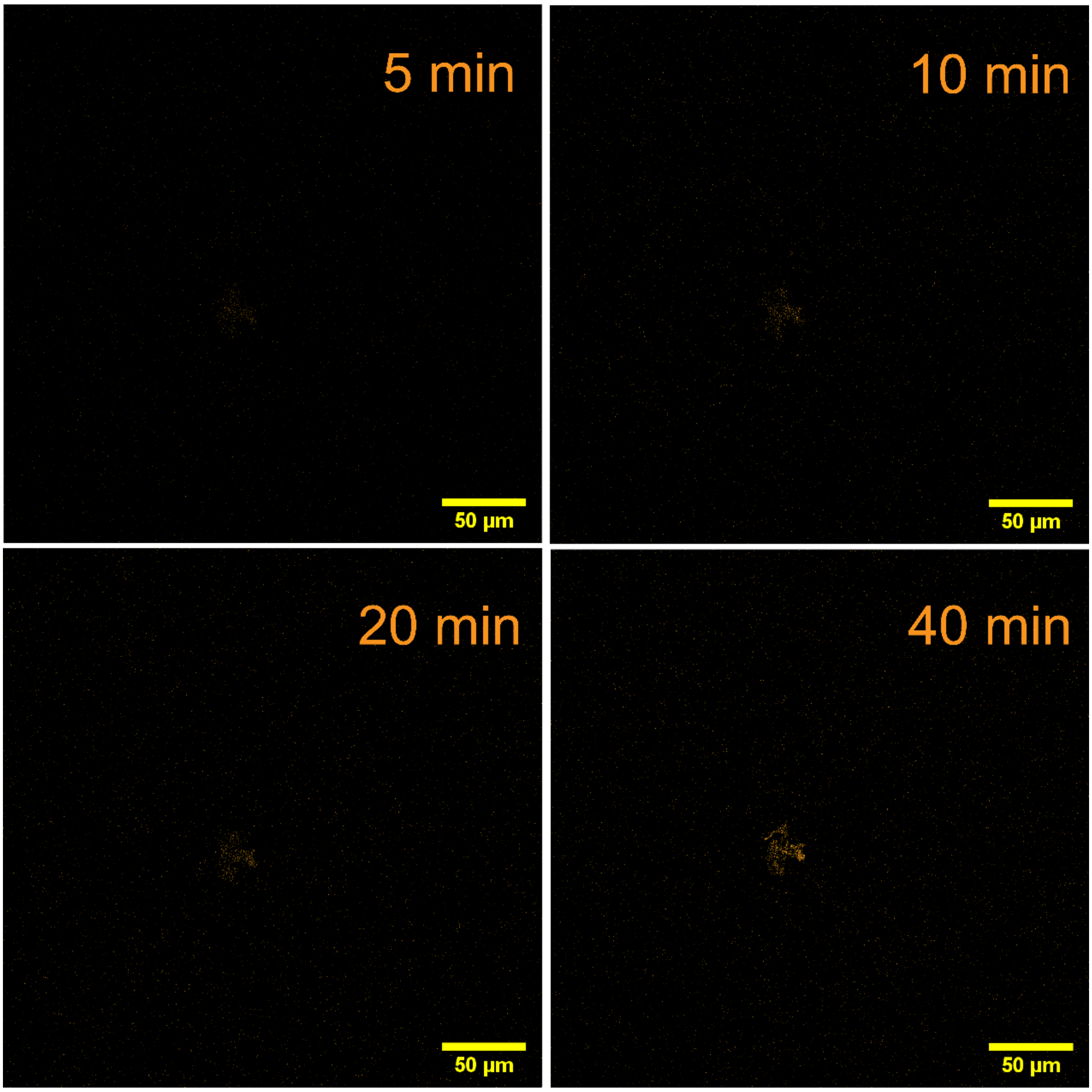

Supplementary Figure 28. Confocal images of SMC-SP-PEI-SRB recovery in dark process in aqueous solution over time. ($[SMC]_{initial}$ = 0.225 mM, [PEI] = 5 μg mL^{-1}, [SRB] = 0.001 mM)

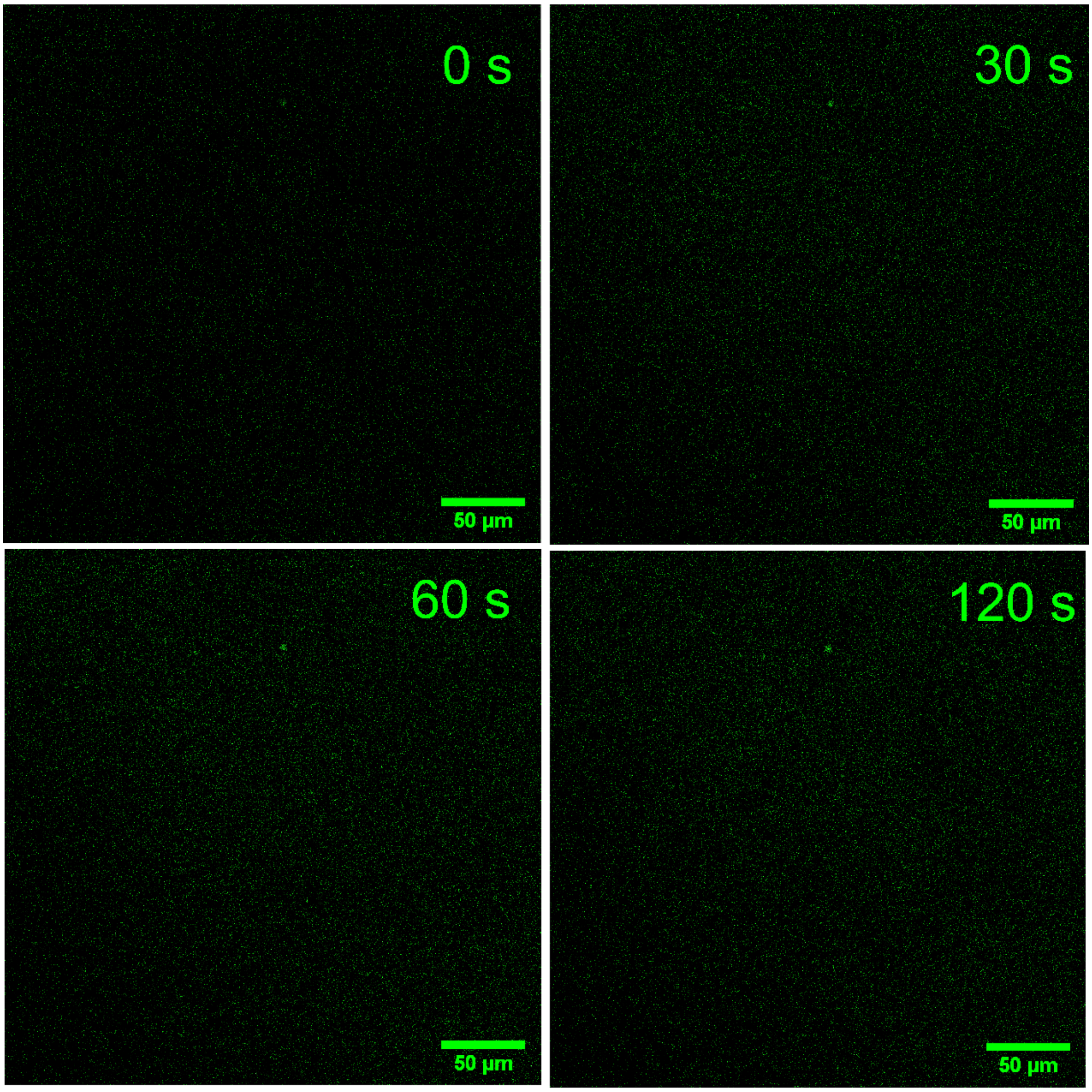

Supplementary Figure 29. Confocal images of SMC-SP-PEI-CFDA irradiation process in aqueous solution over time. ($[SMC]_{initial}$ = 0.225 mM, [PEI] = 5 μg mL^{-1}, [CFDA] = 0.01 mM)

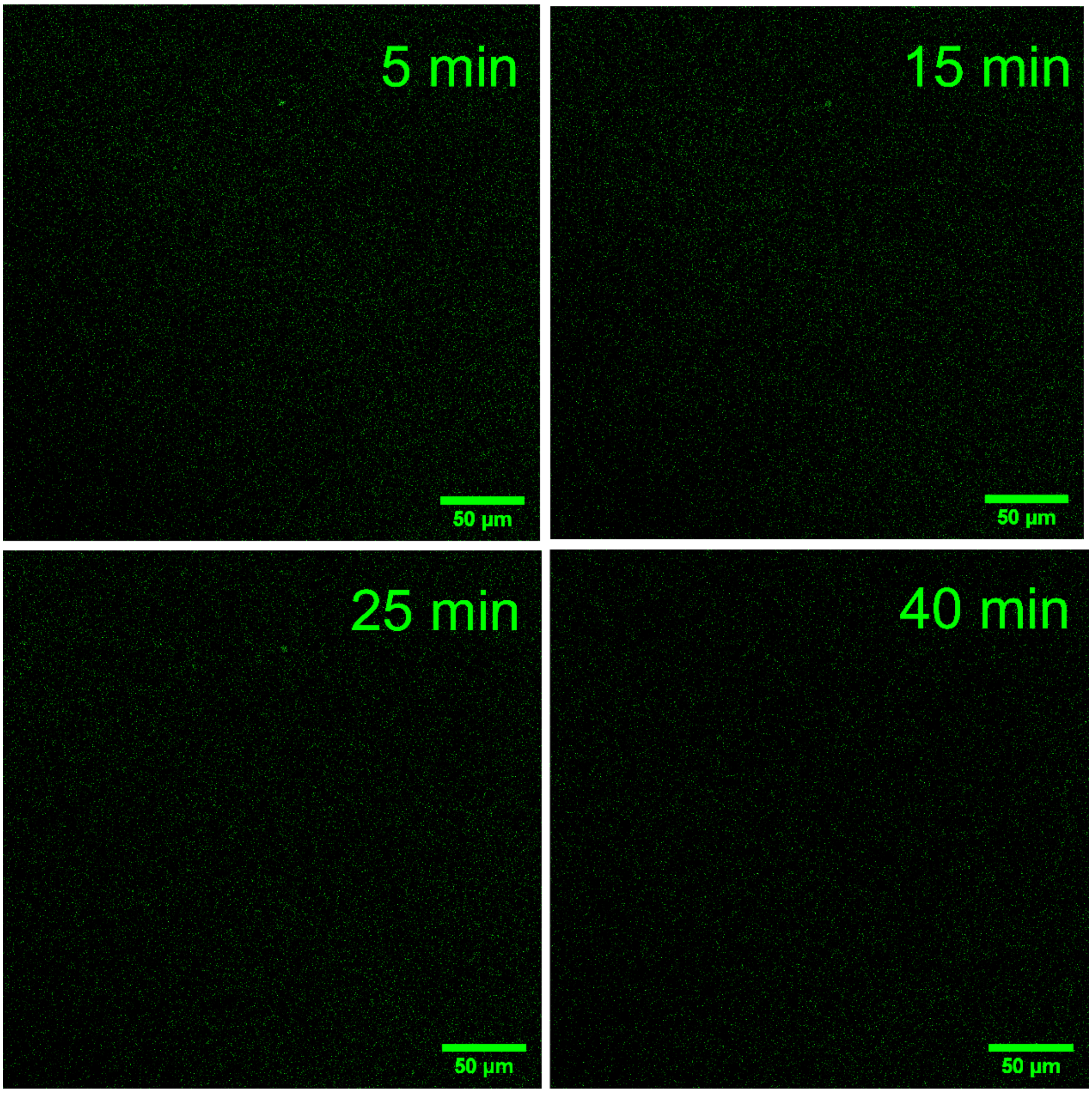

Supplementary Figure 30. Confocal images of SMC-SP-PEI-CFDA recovery in dark process in aqueous solution over time. ($[SMC]_{initial}$ = 0.225 mM, [PEI] = 5 μg mL^{-1}, [CFDA] = 0.01 mM)

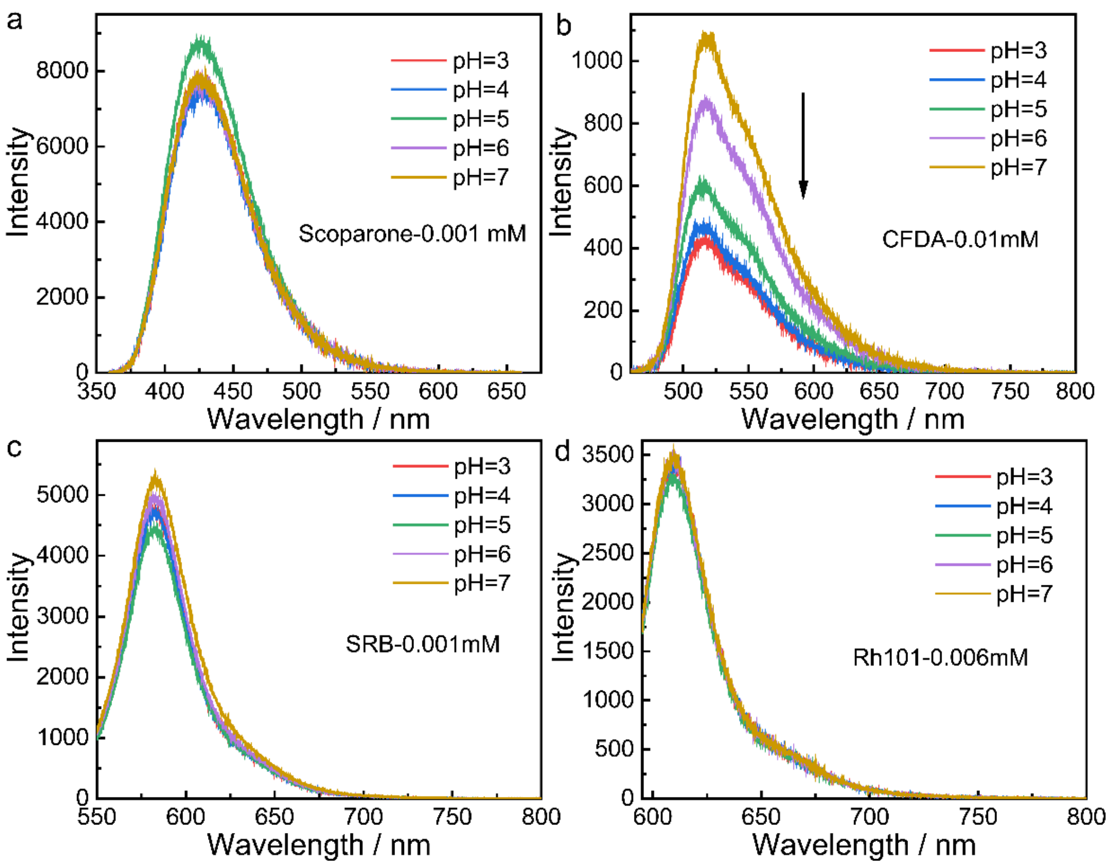

Supplementary Figure 31. (a) Fluorescence intensity of scoparone (0.001 mM) in different pH. (b) Fluorescence intensity of CFDA (0.01 mM) in different pH. (c) Fluorescence intensity of SRB (0.001 mM) in different pH. (d) Fluorescence intensity of Rh101 (0.006 mM) in different pH.

Supplementary Table 1. The quantum yield of the dissipative system loaded with different fluorescent dyes before and after 420 nm irradiation. ($[SMC]_{initial}$ = 0.225 mM, [PEI] = 5 μg mL^{-1}, [Scoparone] = 0.001 mM, [CFDA] = 0.01 mM, [Rh101] = 0.006 mM, [SRB] = 0.001 mM)

Fluorescent dye	In dark	IR right after 420 nm
Scoparone	<0.1%	18.59
CFDA	<0.1%	23.92
Rh101	3	14.17
SRB	15.52	2.75

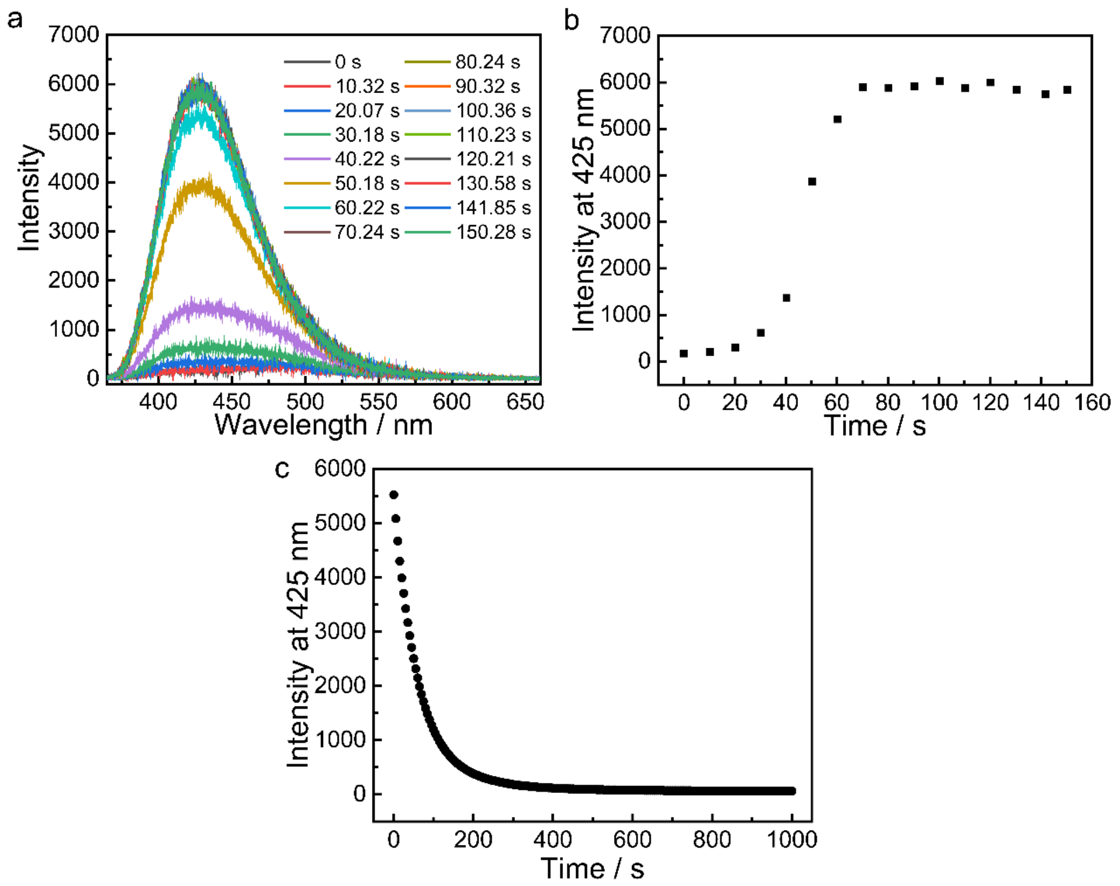

Supplementary Figure 32. (a) Fluorescence spectra of SMC-SP-PEI-scoparone upon irradiation by 420 nm light in aqueous solution with different time length. (b) Fluorescence intensity at 425 nm of (a). (c) Fluorescence intensity at 425 of SMC-SP-PEI-scoparone recover in dark right after irradiation by 420 nm light in aqueous solution with different time length. ($[SMC]_{initial}$ = 0.225 mM, [PEI] = 5 μg mL^{-1}, [Scoparone] = 0.001 mM, λ_{ex} = 340 nm)

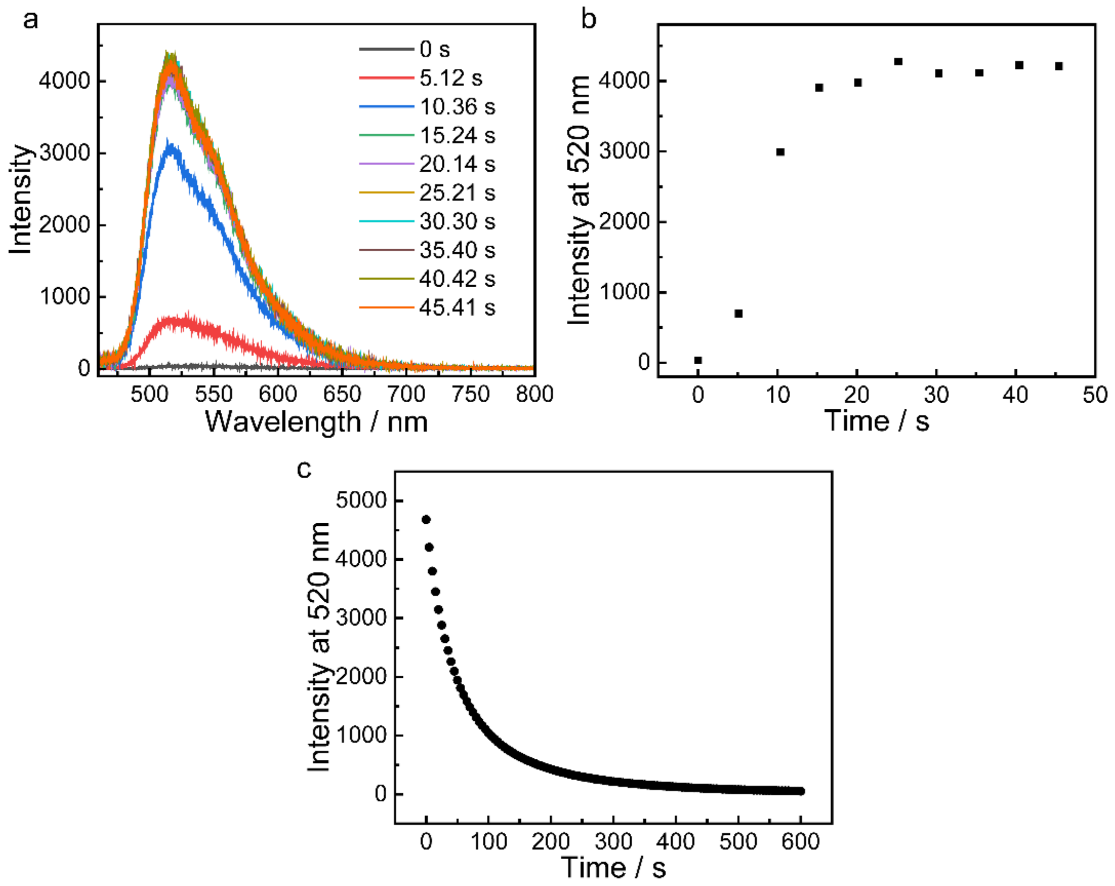

Supplementary Figure 33. (a) Fluorescence spectra of SMC-SP-PEI-CFDA upon irradiation by 420 nm light in aqueous solution with different time length. (b) Fluorescence intensity at 520 nm of (a). (c) Fluorescence intensity at 520 of SMC-SP-PEI-scoparone recover in dark right after irradiation by 420 nm light in aqueous solution with different time length. ($[SMC]_{initial}$ = 0.225 mM, [PEI] = 5 μg mL^{-1}, [CFDA] = 0.01 mM, λ_{ex} = 440 nm)

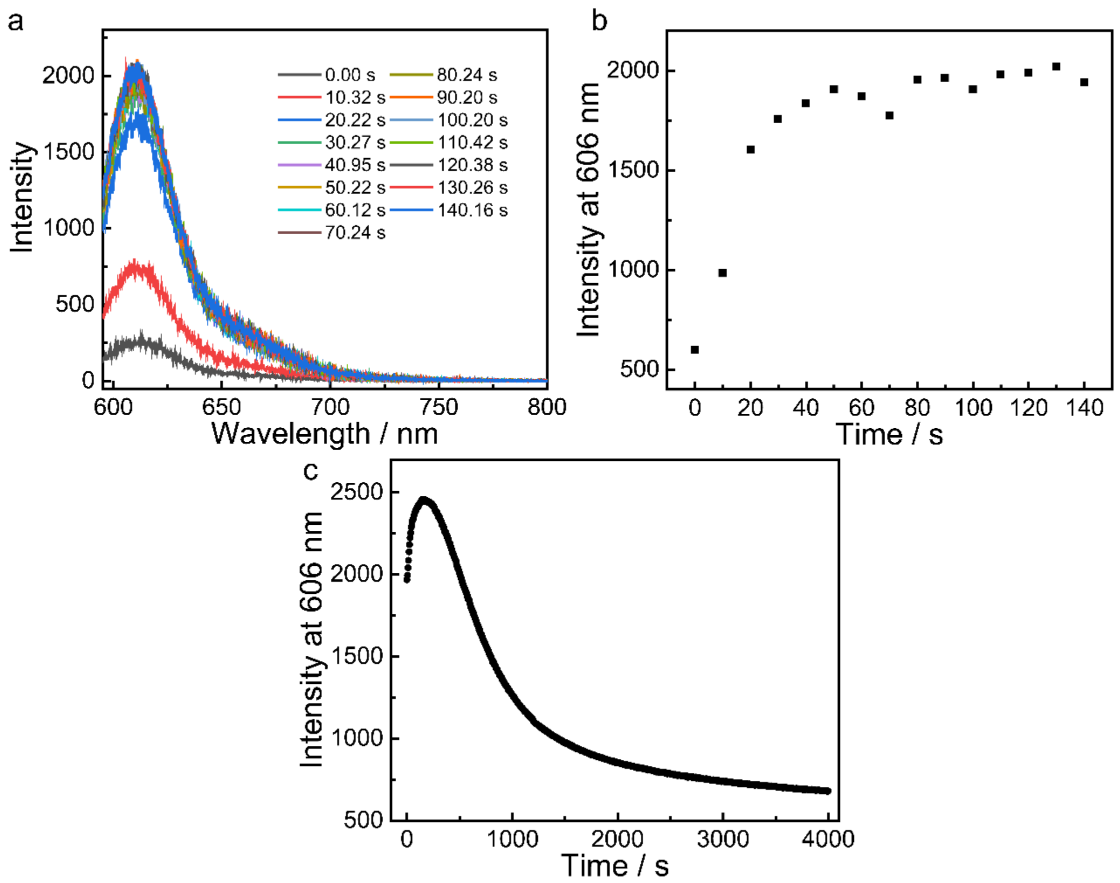

Supplementary Figure 34. (a) Fluorescence spectra of SMC-SP-PEI-Rh101 upon irradiation by 420 nm light in aqueous solution with different time length. (b) Fluorescence intensity at 606 nm of (a). (c) Fluorescence intensity at 606 nm of SMC-SP-PEI-Rh101 recover in dark right after irradiation by 420 nm light in aqueous solution with different time length. ($[SMC]_{initial}$ = 0.225 mM, [PEI] = 5 μg mL^{-1}, [Rh101] = 0.006 mM, λ_{ex} = 585 nm)

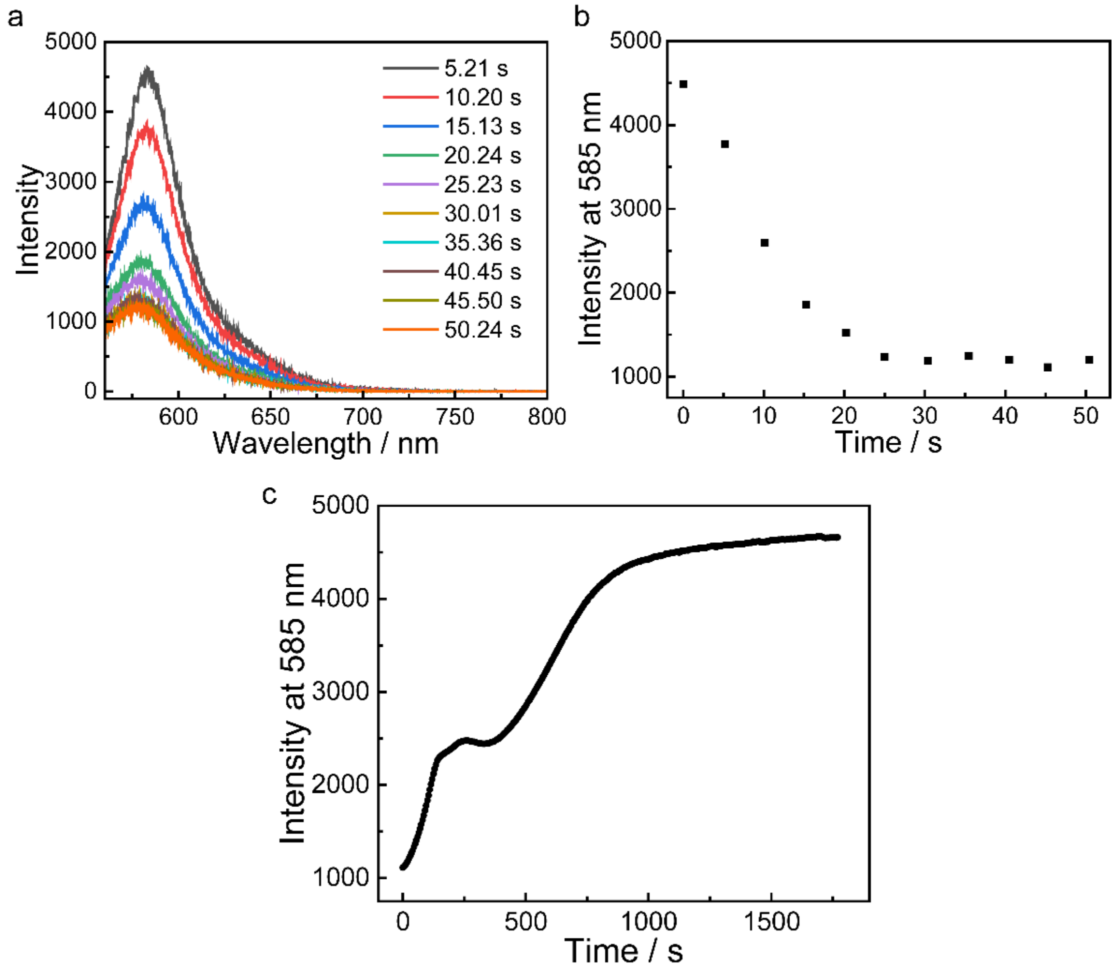

Supplementary Figure 35. (a) Fluorescence spectra of SMC-SP-PEI-SRB upon irradiation by 420 nm light in aqueous solution with different time length. (b) Fluorescence intensity at 585 nm of (a). (c) Fluorescence intensity at 585 nm of SMC-SP-PEI-SRB recover in dark right after irradiation by 420 nm light in aqueous solution with different time length. ($[SMC]_{initial}$ = 0.225 mM, [PEI] = 5 μg mL^{-1}, [SRB] = 0.001 mM, λ_{ex} = 540 nm)

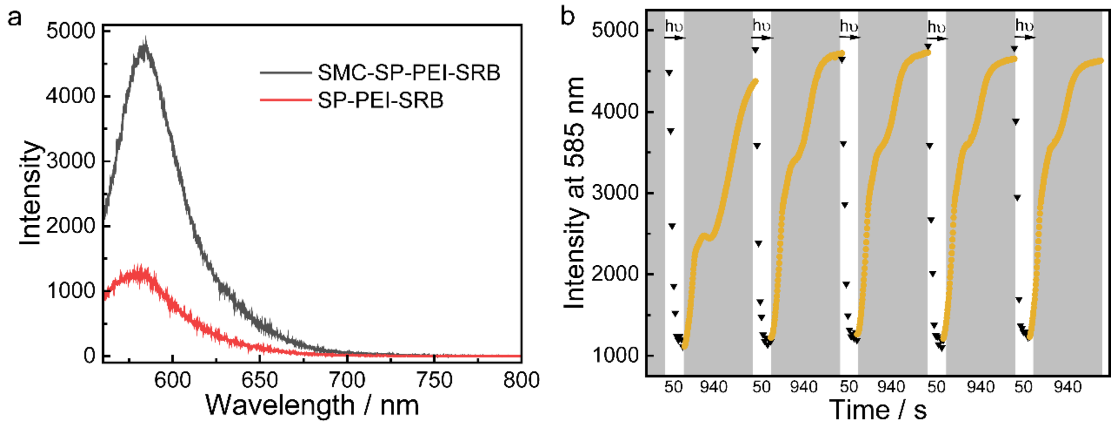

Supplementary Figure 36. (a) Fluorescence emission of SMEH-SP-PEI-SRB and SP-PEI-SRB. (b) Reversible fluorescence variation of SRB at 585 nm for 5 cycles between the SMC-SP-PEI and SP-PEI dissipative process over time. The white parts represent the light-induced formation of ASP-CS and the grey parts represents the dissociation process, respectively. ($[SMC]_{initial}$ = 0.225 mM, [PEI] = 5 μg mL^{-1}, [SRB] = 0.001 mM, λ_{ex} = 560 nm)

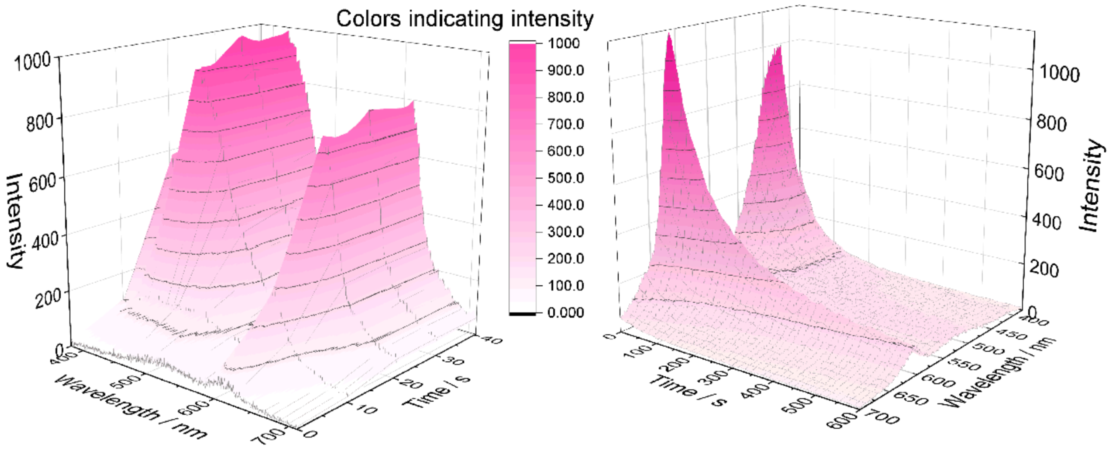

Supplementary Figure 37. 3D fluorescence spectra of light-induced process and thermal deformation process loading scoparone and Rh101. ([Scoparone] = 0.0003 mM, [Rh101] = 0.006 mM) ($[SMC]_{initial}$ = 0.225 mM, [PEI] = 5 μg mL^{-1}).

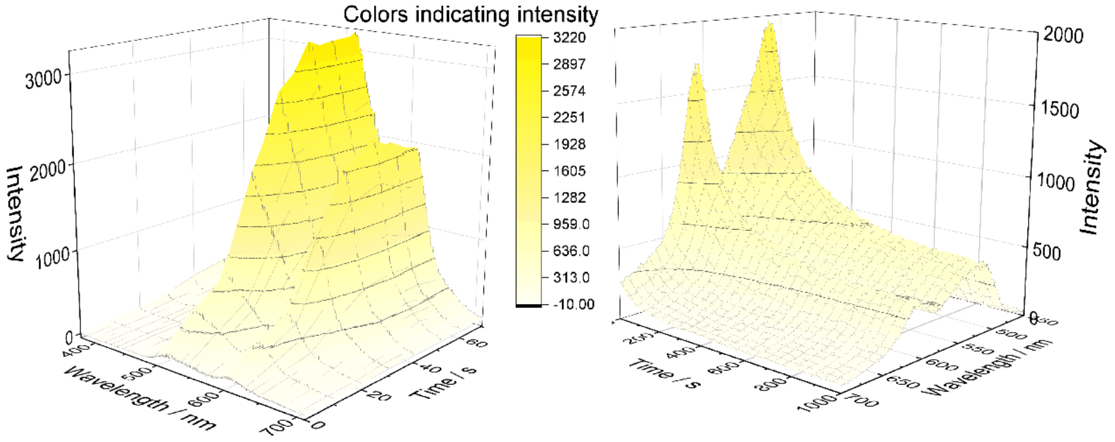

Supplementary Figure 38. 3D fluorescence spectra of light-induced process and thermal deformation process loading CFDA and Rh101. ([CFDA] = 0.002 mM, [Rh101] = 0.003 mM) ($[SMC]_{initial}$ = 0.225 mM, [PEI] = 5 μg mL^{-1}).

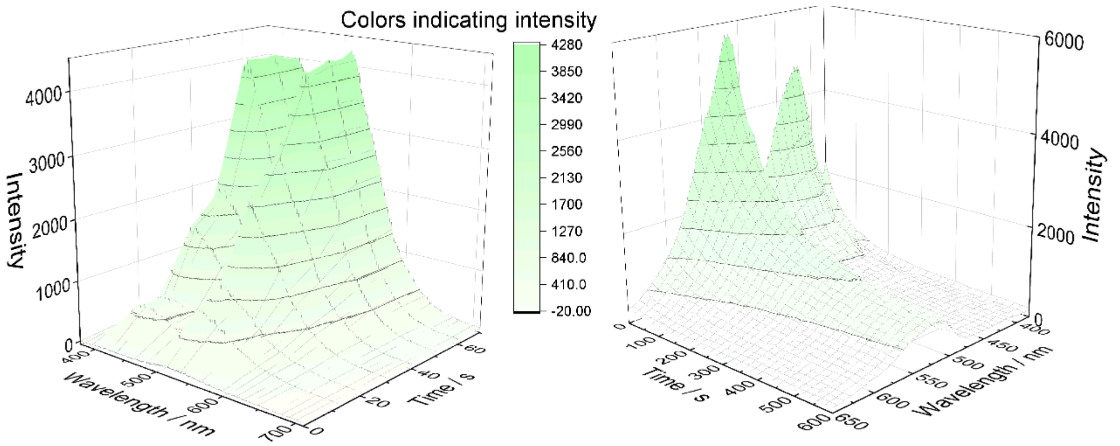

Supplementary Figure 39. 3D fluorescence spectra of light-induced process and thermal deformation process loading scoparone and CFDA. ([Scoparone] = 0.0001 mM, [CFDA] = 0.03 mM) ($[SMC]_{initial}$ = 0.225 mM, [PEI] = 5 μg mL^{-1}).

Section E. Creating self-erasing multicolor fluorescent images

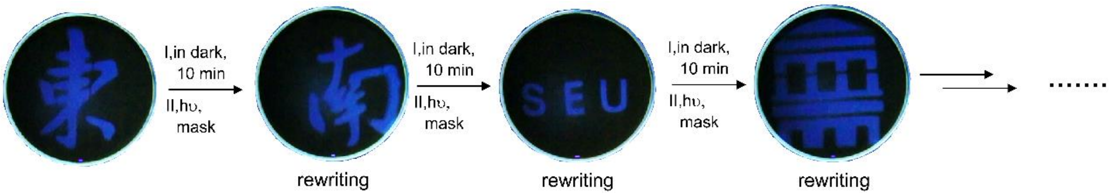

Supplementary Figure 40. The initial status of the assembly system loading scoparone under 365 nm right after 420 nm irradiation and replacing different masks.

Section F. ^{1}H-NMR, ^{13}C-NMR and HRMS spectra

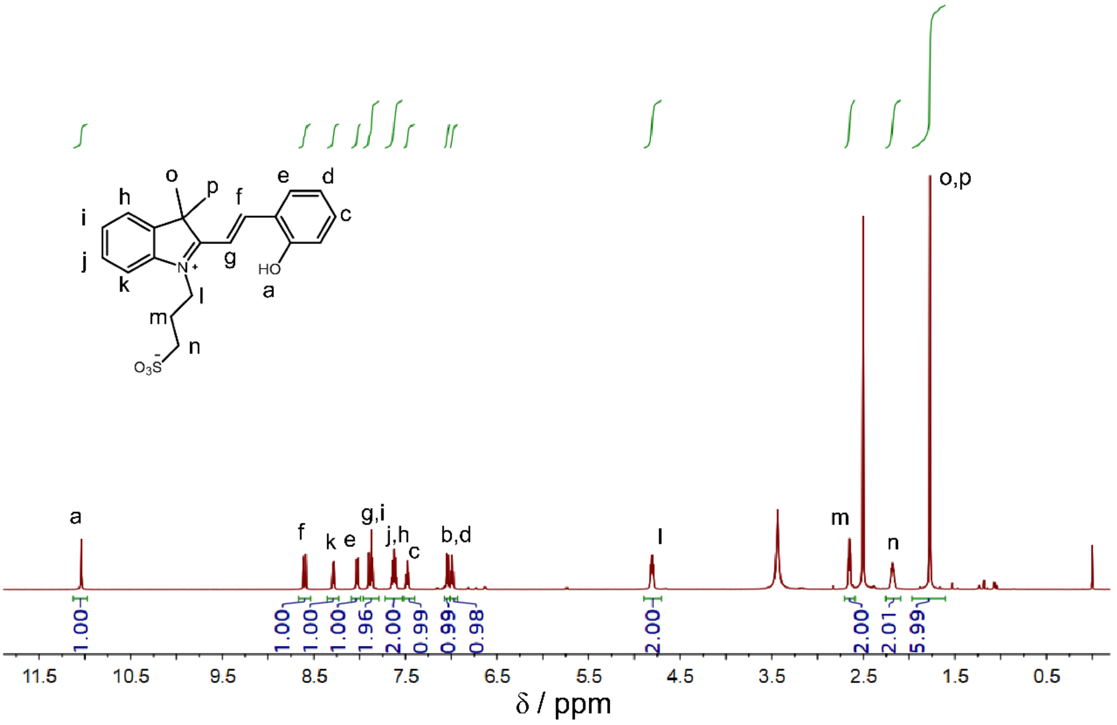

Supplementary Figure 41. ^{1}H-NMR spectra (600 MHz, DMSO-d_6) of SMC.

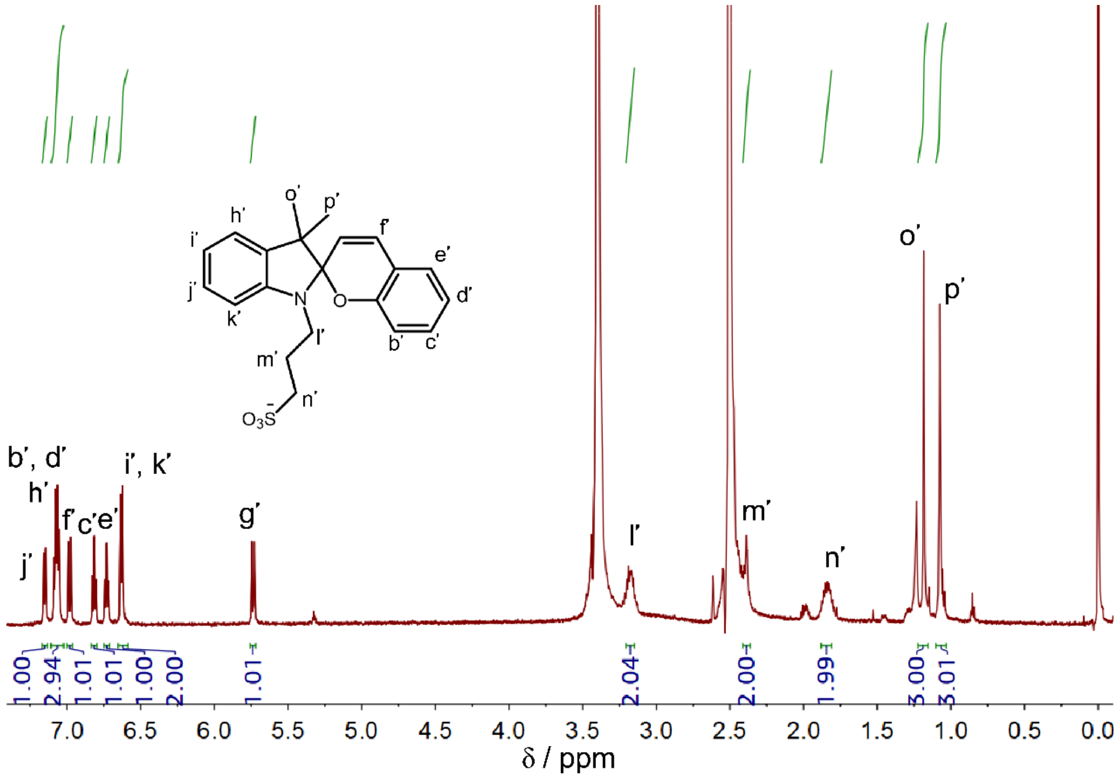

Supplementary Figure 42. ^{1}H-NMR spectra (600 MHz, DMSO-d_6) of SP.

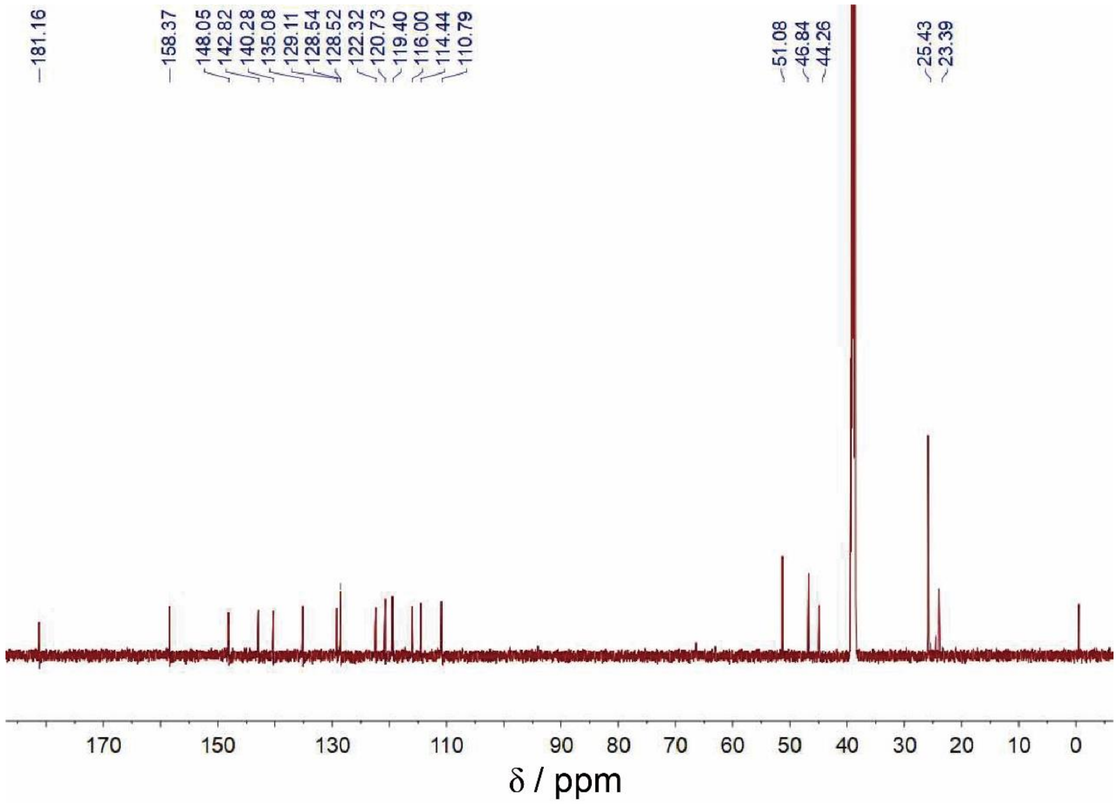

Supplementary Figure 43. ^{13}C-NMR spectra (151 MHz, DMSO-d_6) of SMC.

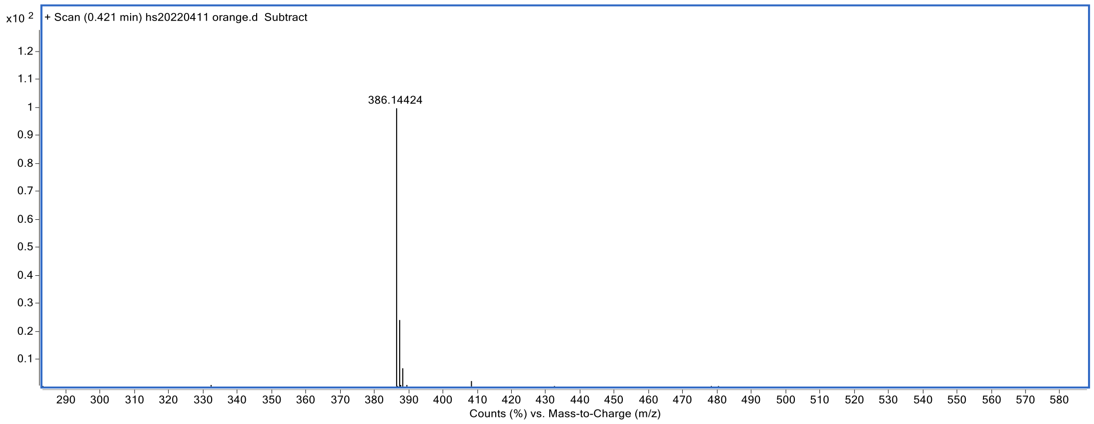

Supplementary Figure 44. HRMS spectra of SMC.

References

1. Chen, X. M., Hou, X. F., Bisoyi, H. K., Feng, W. J., Cao, Q., Huang, S., Yang, H., Chen, D. & Li, Q. Light-fueled transient supramolecular assemblies in water as fluorescence modulators. *Nat. Commun.* **12**, 4993 (2021).
