# Supplementary material for: Light-activated photodeformable supramolecular dissipative self-assemblies
Source: Nat Commun. 2022 Jun 9;13:3216. doi: 10.1038/s41467-022-30969-2 (PMC9184535; doi:10.1038/s41467-022-30969-2)
Supplement: Supplementary file 2 — Description of Additional Supplementary Files [file 41467_2022_30969_MOESM2_ESM.pdf]

Description of Additional Supplementary Files

File Name: Supplementary Movie 1
Description: The confocal images and fluorescent particle counts of light-irradiation and thermal relaxation of the photodeformable dissipative supramolecular self-assemblies by loading SRB.

File Name: Supplementary Movie 2
Description: The confocal images and fluorescent particle counts of light-irradiation and thermal relaxation of the photodeformable dissipative supramolecular self-assemblies by loading CFDA.

File Name: Supplementary Movie 3
Description: Dynamic fluorescent variation of loaded scoparone over time during thermal relaxation.

File Name: Supplementary Movie 4
Description: Dynamic fluorescent variation of loaded CFDA over time during thermal relaxation.

File Name: Supplementary Movie 5
Description: Dynamic fluorescent variation of loaded Rh101 over time during thermal relaxation.
